# m^6^A demethylase ALKBH5 attenuates doxorubicin-induced cardiotoxicity via posttranscriptional stabilization of Rasal3

**DOI:** 10.1016/j.isci.2023.106215

**Published:** 2023-02-16

**Authors:** Ri-Feng Gao, Kun Yang, Ya-Nan Qu, Xiang Wei, Jia-Ran Shi, Chun-Yu Lv, Yong-Chao Zhao, Xiao-Lei Sun, Ying-Jia Xu, Yi-Qing Yang

**Affiliations:** 1 Department of Cardiology, Shanghai Fifth People’s Hospital, Fudan University, Shanghai 200240, China; 2 Department of Cardiology, Shanghai Institute of Cardiovascular Diseases, Zhongshan Hospital, Fudan University, Shanghai 200232, China; 3 Department of Cardiology, the First Affiliated Hospital, Zhejiang University, Hangzhou 310003, China; 4 Shenzhen Key Laboratory for Neuronal Structural Biology, Biomedical Research Institute, Shenzhen Peking University-The Hong Kong University of Science and Technology Medical Center, Shenzhen 200240, China; 5 Department of Cardiovascular Research Laboratory, Shanghai Fifth People’s Hospital, Fudan University, Shanghai 518036, China; 6 Department of Central Laboratory, Shanghai Fifth People’s Hospital, Fudan University, Shanghai 200240, China

**Keywords:** Biological sciences, Pathophysiology, Molecular biology

## Abstract

The clinical application of anthracyclines such as doxorubicin (DOX) is limited due to their cardiotoxicity. N6-methyladenosine (m^6^A) plays an essential role in numerous biological processes. However, the roles of m^6^A and m^6^A demethylase ALKBH5 in DOX-induced cardiotoxicity (DIC) remain unclear. In this research, DIC models were constructed using *Alkbh5*-knockout (KO), *Alkbh5*-knockin (KI), and *Alkbh5*-myocardial-specific knockout (ALKBH5^flox/flox, αMyHC−Cre^) mice. Cardiac function and DOX-mediated signal transduction were investigated. As a result, both *Alkbh5* whole-body KO and myocardial-specific KO mice had increased mortality, decreased cardiac function, and aggravated DIC injury with severe myocardial mitochondrial damage. Conversely, ALKBH5 overexpression alleviated DOX-mediated mitochondrial injury, increased survival, and improved myocardial function. Mechanistically, ALKBH5 regulated the expression of Rasal3 in an m^6^A-dependent manner through posttranscriptional mRNA regulation and reduced *Rasal3* mRNA stability, thus activating RAS3, inhibiting apoptosis through the RAS/RAF/ERK signaling pathway, and alleviating DIC injury. These findings indicate the potential therapeutic effect of ALKBH5 on DIC.

## Highlights

• *Alkbh5*-null mice had increased mortality due to doxorubicin-induced cardiotoxicity
• ALKBH5 overexpression alleviated doxorubicin-induced cardiotoxicity
• ALKBH5 regulated the expression of Rasal3 in an m6A-dependent manner
• ALKBH5 reduced *Rasal3* mRNA stability

## Graphical abstract

**Figure:**
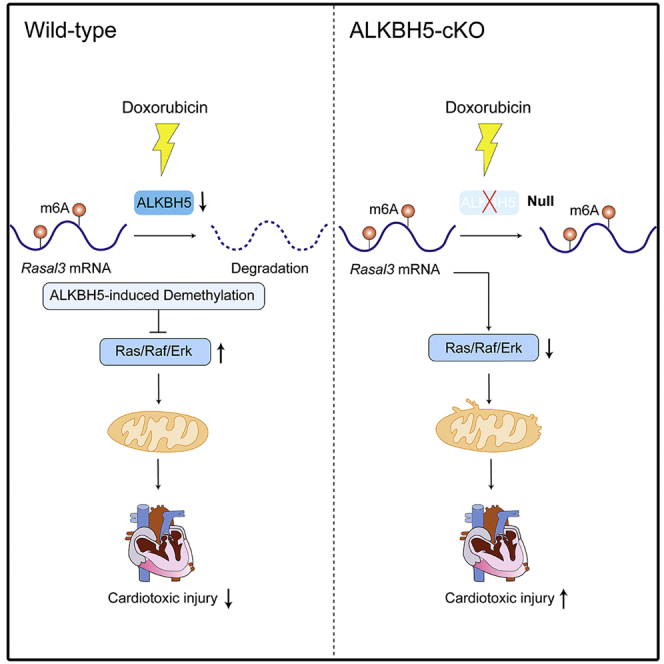

## Introduction

Anthracyclines, such as doxorubicin (DOX), are cytotoxic antibiotics that are widely used as anticancer drugs.^1^^,^^2^^,^^3^^,^^4^ They inhibit the proliferation of cancer cells but also cause pathological changes in the myocardium; promote apoptosis, necrosis, and pyroptosis of myocardial cells; and damage myocardial mitochondria.^5^^,^^6^^,^^7^^,^^8^^,^^9^^,^^10^^,^^11^ The major adverse effects of DOX that limit its clinical utility are cardiovascular toxicities: hypotension, tachycardia, arrhythmias, and ultimately congestive heart failure.^5^^,^^12^^,^^13^^,^^14^^,^^15^ The treatment of DOX-induced cardiotoxicity (DIC) has always been a focus of clinical research and remains challenging. Therefore, there is a great need to identify underlying mechanisms, and new treatment strategies for DIC.

N^6^-methyladenosine (m^6^A) refers to methylation modification of the N atom at position 6 of adenosine, which is the most common form of posttranscriptional modification in mammals.^16^^,^^17^^,^^18^ The methylation of m^6^A is reversible and is regulated by enzymes including methyltransferases (“writers”), demethylases (“erasers”), and methylation-reading proteins (“readers”).^17^^,^^19^^,^^20^ Recent studies have shown that the m^6^A demethylase ALKB homolog 5 (ALKBH5) is involved in the repair of damages induced by cardiovascular diseases.^21^^,^^22^ However, the role of ALKBH5 in DIC has not been reported.

Herein, we generated murine DIC models to show that ALKBH5 plays an important role in protecting myocardial mitochondria and cardiomyocyte (CM) survival in DIC through m^6^A demethylation. Furthermore, ALKBH5 disrupts Rasal3 mRNA stability in CMs and alters the Ras/Raf/Erk signaling.

## Results

### DOX-induced cardiotoxic injury and downregulation of ALKBH5 expression in myocardial tissue

**Figure 1:**
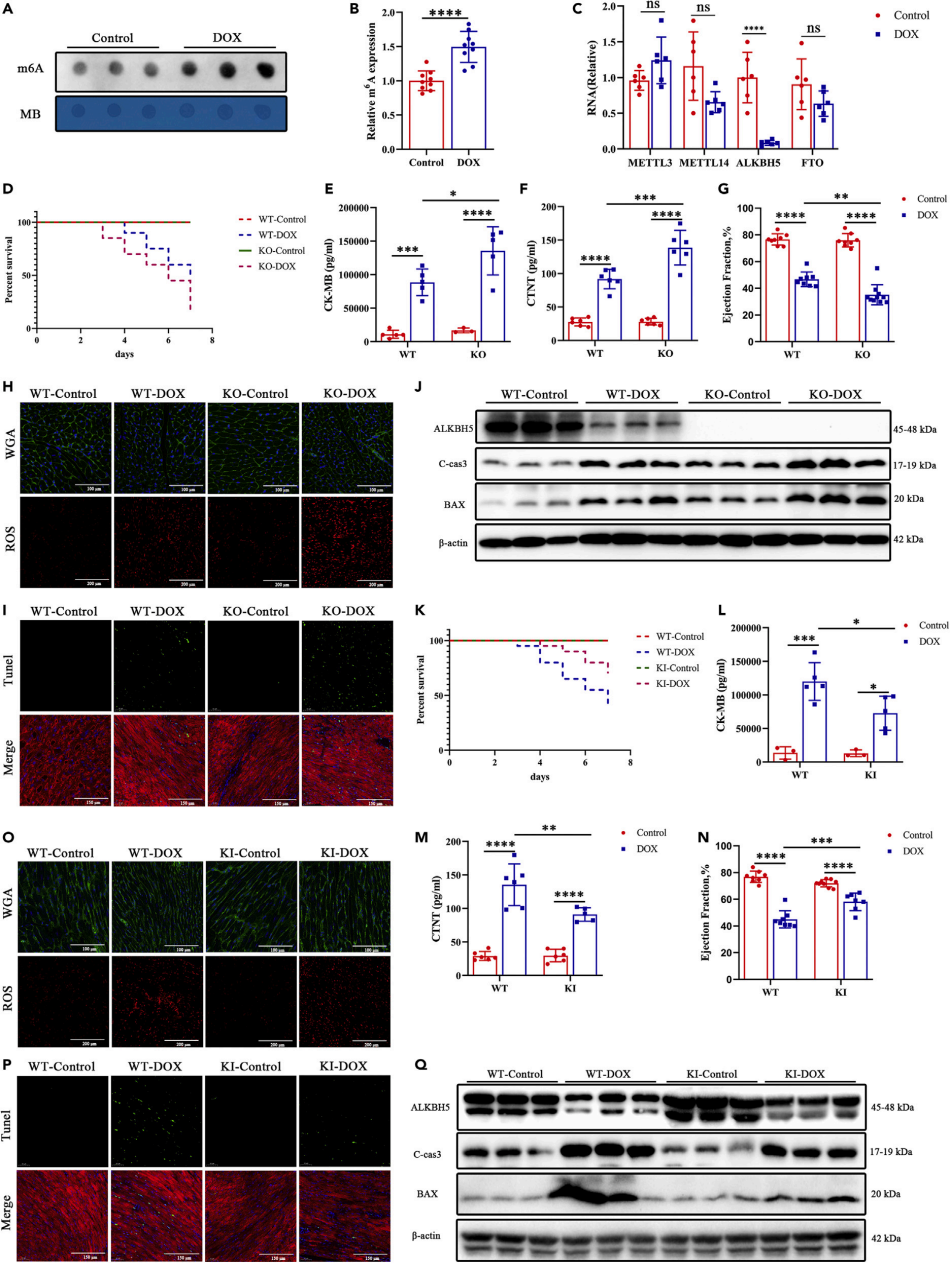
ALKBH5-KO aggravates DIC, and ALKBH5-KI attenuates DIC injury (A and B) Representative dot blot images showing m6A abundance after injection under normal saline (control) and DOX (n > 6). (C) RT-qPCR analysis of the mRNA expression levels of METTL3, METTL14, ALKBH5, and FTO in DIC mice (n = 6). (D) Kaplan-Meier survival curves showing the survival of DOX-stressed mice after ALKBH5-knockout (KO) under normal saline (control) and DOX (n = 20). Detection of cardiotoxicity indexes CK-MB (E; n = 5) and CTnT (F; n = 6) by ELISA. (G) Ejection fraction (n > 6). (H) WGA (Bar = 100 μm) and ROS (Bar = 200 μm) staining of ALKBH5-KO and WT mice in DIC model. (I) Apoptosis measured by TUNEL staining in ALKBH5-KO control and ALKBH5-KO heart sections (Bar = 150 μm). (J) Western blot analysis of the protein expression levels of ALKBH5, cleaved caspase-3, and BAX levels in DIC. (K) Kaplan-Meier survival curves showing the survival of DOX-stressed mice after ALKBH5-knockin (KI) under normal saline and DOX. Detection of cardiotoxicity indexes CK-MB (L) and CTnT (M) by ELISA. (N) Ejection fraction of ALKBH5-KI and WT mice. (O) WGA (Bar = 100 μm) and ROS (Bar = 200 μm) staining of ALKBH5-KI and WT mice in DIC model. (P) Apoptosis measured by TUNEL staining in WT and ALKBH5-KI heart sections (Bar = 150 μm). (Q) Western blot analysis of the apoptosis protein expression levels of ALKBH5, cleaved caspase-3, and BAX levels in ALKBH5-KI-DOX and WT-DOX. Data are depicted as the mean ± SEM. Statistical significance was determined by one-way ANOVA with a post-hoc Holm-Sidak test. Here, ns, not significant; ∗p < 0.05; ∗∗p < 0.05; ∗∗∗p < 0.001; ∗∗∗∗p < 0.0001.

We constructed a murine DIC model (Figure S1A) to assess myocardial function and regulation of m^6^A-related enzymes. We found that m^6^A methylation increased (Figures 1A and 1B), while ALKBH5 expression was decreased (Figure 1C) in mice with DIC. Survival was significantly decreased by DOX treatment (100% vs 40%; log rank test; p < 0.0001; Figure S2A). Both body (Figure S2B) and heart (Figure S2C) weights decreased, while the expression of the markers of myocardial toxic injury, CK-MB (Figure S2D) and cTnT (Figure S2E), significantly increased. Echocardiography showed that both myocardial left ventricular ejection fraction (LVEF) and left ventricular fractional shortening (LVFS) were decreased in mice with DIC (Figures S2F and S2G). Single-cell contractility assays showed no differences in CM resting lengths in DIC mice (Figure S2H). Regarding CM systolic function, peak shortening (Figure S2K), the maximal velocity of shortening (−dL/dt; Figure S2J), and time to peak strain (Figure S2L) were significantly lower in DIC than in control mice. Regarding CM diastolic function, the maximal velocity of re-lengthening (+dL/dt; Figure S2I) was significantly decreased in DIC mice, but there was no significant difference in the time to 90% re-lengthening (Figure S2M) between DIC DOX-treated and control mice. This indicates that CM contractility decreases in DIC mice. Wheat germ agglutinin (WGA) and reactive oxygen species (ROS) staining showed that CMs were made smaller by DOX treatment (Figures S2N and S2O) relative to controls, while oxidative stress injury was aggravated (Figure S2O). Western blotting (Figure S2Q) and terminal deoxynucleotidyl transferase dUTP nick-end labeling (TUNEL) staining (Figure S2P) showed that the myocardial apoptosis was increased by DOX treatment.

Next, we assessed the effect of DOX on myocardial mitochondrial function. As shown in Figure S3A, in contrast to controls treated with saline, mice treated with DOX exhibited cardiac ultrastructural defects and mitochondria with disrupted cristae. We found that myocardial mitochondrial JC-1 was decreased by 18.92% by DOX treatment (Figures S3B and S3C). CM respiration was markedly decreased by DOX treatment (Figures S3D and S3E). These data demonstrate that DOX treatment affects myocardial mitochondrial function to decrease CM ATP generation (Figures S3D and S3F), inducing myocardial injury, which may play a role in regulating m^6^A levels through ALKBH5.

### ALKBH5 knockout (KO) aggravates while ALKBH5 knockin (KI) attenuates DIC injury

Following DOX treatment, *Alkbh5*-KO survival was significantly decreased compared with that of controls (log rank test; p = 0. 0.0335; Figure 1D). Both body (Figure S4A) and heart (Figure S4B) weights decreased, while expression of the cardiotoxicity markers, CK-MB (Figure 1E) and cTnT (Figure 1F), were significantly increased by DOX treatment. Echocardiography showed that both LVEF (Figure 1G) and LVFS (Figures S4C–S4E) were decreased by DOX treatment. Single-cell contractility assays showed that DOX decreased systolic function, while there were no significant differences in diastolic function between groups (Figures S4F–S4K). WGA and ROS staining showed that DOX treatment decreased CM size and that ROS injury was aggravated (Figures 1H, S4M and S4N), respectively. TUNEL staining (Figures 1I and S4Q) and apoptotic protein expression (Figures 1J, S4O and S4P) showed that apoptosis was significantly elevated by DOX treatment. Taken together, these findings suggest that DOX treatment aggravates myocardial injury and increases mortality in ALKBH5-deficient mice.

We next constructed *Alkbh5*-KI mice as a DIC model. Their survival was significantly increased over that of wild-type (WT) mice (70 vs. 40%, respectively; log rank test; p = 0.0443; Figure 1K). Both body (Figure S5A) and heart (Figure S5B) weights were increased, while CK-MB (Figure 1L) and cTnT (Figure 1M) levels were significantly decreased in *Alkbh5*-KI mice. Systolic function in KI mice treated with DOX was significantly improved over that of controls (Figures 1N and S5C–S5K). WGA and ROS staining (Figures 1O, S5M and S5N) showed that KI CMs were larger and that ROS injury was decreased relative to controls. Apoptotic protein expression was upregulated, and the number of apoptotic cells increased in DOX-treated KI mice (Figures 1P, 1Q, and S5O–S5Q).

We found that DOX treatment decreased myocardial ALKBH5 expression (Figures 1J, 1Q, S4N, and S4O), which in turn resulted in decreased myocardial m^6^A levels, whereas it was difficult to alter m^6^A levels in ALKBH5-deficient mice (Figure S6A). Interestingly, the basal modified level of m^6^A in the myocardium of the *Alkbh5*-KI mice was reduced, while DOX increased myocardial m^6^A in KI mice (Figure S6B).

### Myocardial-specific ALKBH5 KO aggravates DIC and chronic DIC (CDIC) injury

**Figure 2:**
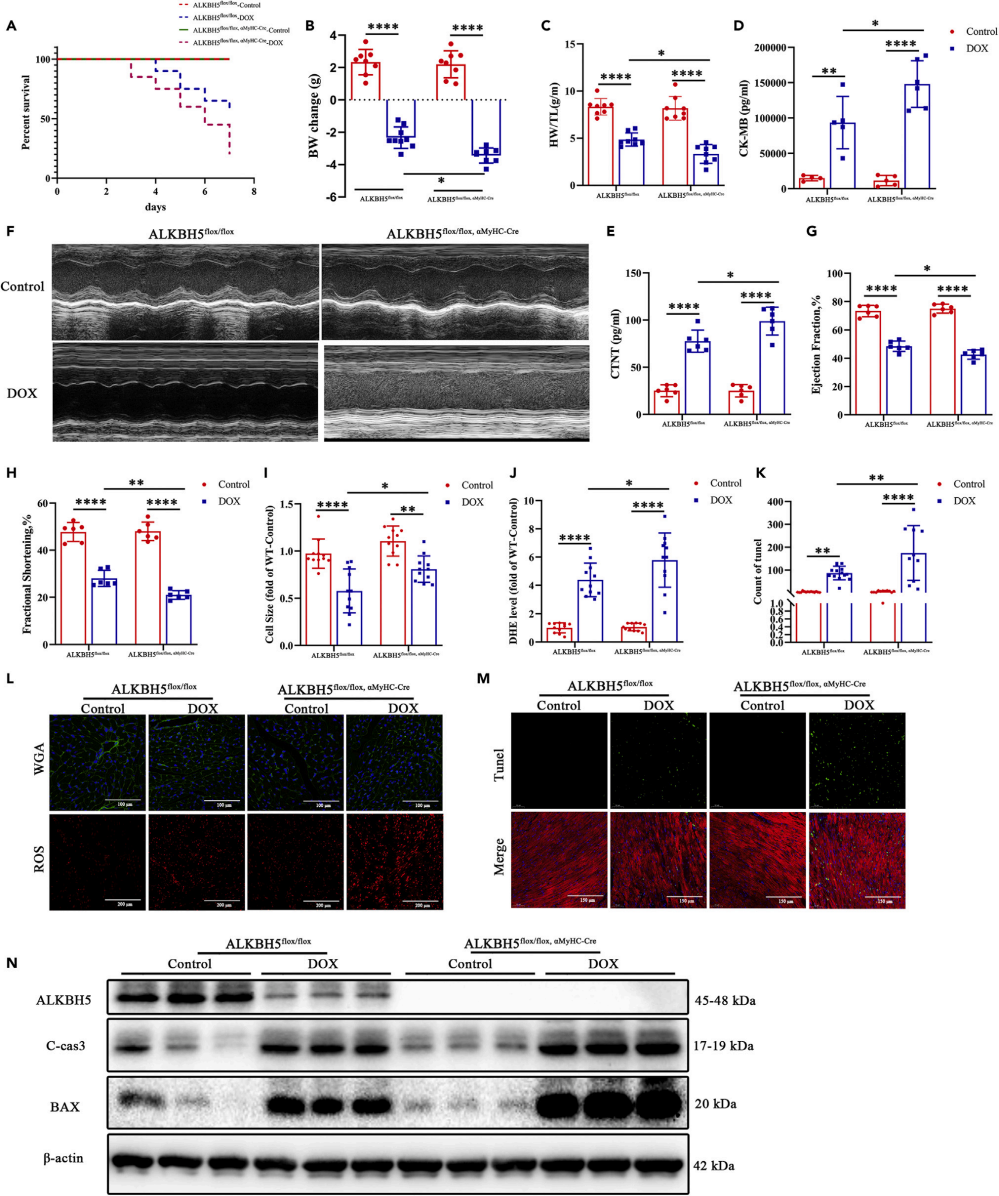
ALKBH5-myocardial-specific knockout aggravates DIC injury (A) Kaplan-Meier survival curves showing the survival of DOX-stressed mice after ALKBH5-myocardial-specific knockout (ALKBH5^flox/flox, α−MyHC-Cre^; n = 20). (B) Body weight change during the DIC experiment in ALKBH5^flox/flox, α−MyHC-Cre^ and control mice (n > 6). (C) Ratio of heart weight to tibial length after DIC (n > 6). Detection of cardiotoxicity indexes CK-MB (D; n = 6) and CTnT (E; n = 6) by ELISA. (F) Representative images of echocardiography tracing in DIC mice. (G) Ejection fraction (n = 6). (H) Fractional shortening (n = 6). WGA (I and L; Bar = 200 μm; n > 6) and ROS (J and L; Bar = 100 μm; n > 6) staining of ALKBH5^flox/flox^ and ALKBH5^flox/flox, α−MyHC-Cre^ mice in DIC model. (K and M) Apoptosis measured by TUNEL staining in ALKBH5^flox/flox^ and ALKBH5^flox/flox, α−MyHC-Cre^ heart sections (Bar = 50 μm; n > 6). (N) Western blot analysis of the protein expression levels of ALKBH5, cleaved caspase-3, and BAX levels in DIC. Data are depicted as the mean ± SEM. Statistical significance was determined by one-way ANOVA with a post-hoc Holm-Sidak test. Here, ns, not significant; ∗p < 0.05; ∗∗p < 0.01; ∗∗∗∗p < 0.0001.

Considering the pivotal role of ALKBH5 in DIC suggested by the whole-mouse KO and KI models, we constructed a cardiomyocyte-specific KO of *Alkbh5* (*Alkbh5*^*flox/flox,*^ ^α−MyHC-Cre^). These mice exhibited decreased survival (55 vs. 20%; log rank test; p = 0.0323; Figure 2A) and decreased body (Figure 2B) and heart (Figure 2C) weights. They also had a diminished cardiac function. In particular, the expression of the injury markers CK-MB (Figure 2D) and cTnT (Figure 2E) were significantly elevated, whereas LVEF (Figures 2F–2H), LVFS (Figure S7A), and contractility of adult myocardial single cells (Figures 2B–2G) were decreased. WGA (Figures 2I and 2L) staining showed that CMs were smaller and ROS (Figures 2J and 2L) staining decreased in DOX-treated *Alkbh5*^*flox/flox*, α−MyHC-Cre^ mice, whereas ROS injury was aggravated. CM apoptosis (Figures 2M, 2N, 2K, S7I and S7J) and m^6^A levels (Figure S7K) in DOX-treated KO mice were significantly increased. Taken together, these results show that the function of ALKBH5 in DIC is intrinsic to CMs.

We next constructed a CDIC model in *Alkbh5*^*flox/flox,*^ ^α−MyHC-Cre^ mice, intraperitoneally injecting 5 mg/kg DOX once weekly for 4 weeks. Echocardiography showed that in CDIC mice, compared with *Alkbh5*^*flox/flox*^ controls lacking Cre, there were no significant changes in early cardiac function, but after 12 weeks, both LVEF and LVFS significantly decreased (Figures S8A–S8D). Compared with controls, DOX-treated *Alkbh5*^*flox/flox*, α−MyHC-Cre^ mice showed no significant differences in body weights (Figure S8E), although their heart weights decreased (Figure S8F) and myocardial apoptosis increased (Figures S8G–S8J). Notably, m^6^A levels were elevated in *Alkbh5*^*flox/flox*,α−MyHC-Cre^ mice (Figure S8K). In conclusion, both DIC and CDIC models show significant aggravation of cardiotoxic injury and decreased myocardial function, suggesting that ALKBH5 acts through CMs.

### ALKBH5 KO aggravates while ALKBH5 overexpression alleviates DOX-induced cardiomyocyte dysfunction

We next created a model by culturing adult CMs from *ALKBH5* KO, KI, and WT control mice and subjecting them to DOX treatment. WT CMs were treated with 1 μM DOX; then, myocardial apoptosis, as well as m^6^A levels and expression of methylation-regulating enzymes, was measured over time. Apoptosis significantly increased after 6 h of DOX treatment (Figures S9A–S9E). Expression of ALKBH5 fluctuated, first increasing and then decreasing (Figures S9A and S9B). However, m^6^A levels were significantly elevated (Figure S9F), whereas the expression of ALKBH5 demethylase was altered, suggesting that ALKBH5 is a critical regulator of m^6^A levels in DOX-induced CM dysfunction.

**Figure 3:**
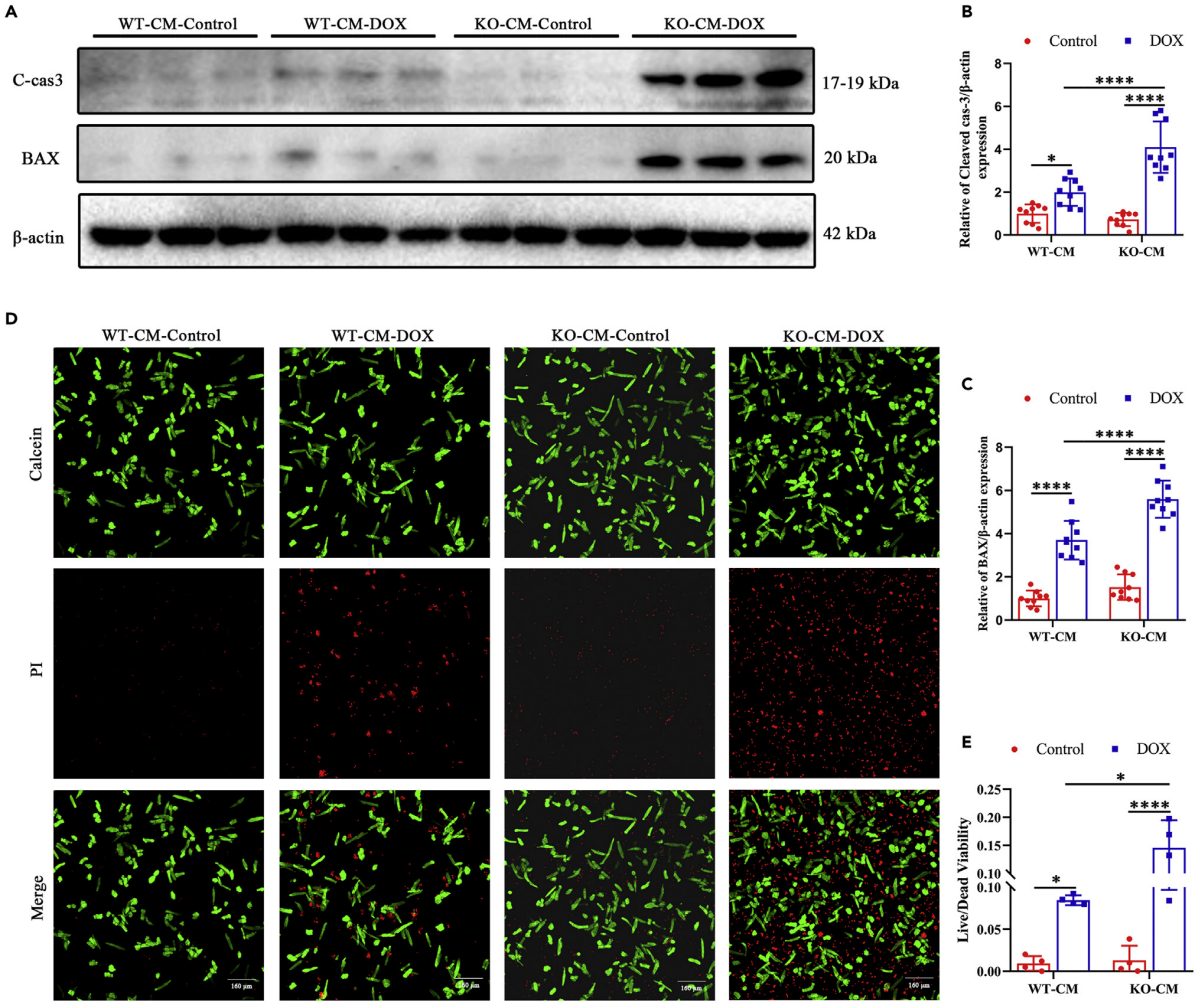
ALKBH5 knockdown aggravates DOX-induced cardiomyocyte dysfunction Western blot analysis of the apoptosis protein expression levels of cleaved caspase-3 (A and B) and BAX (A and C) levels in ALKBH5-KO-CM- and WT-CM after DOX treatment (n > 6). (D and E) Calcein/PI-Live/Dead staining in ALKBH5-KO-CM- and WT-CM after DOX treatment (Bar = 160 μm; n = 4). Data are depicted as the mean ± SEM. Statistical significance was determined by Student’s *t* test, one-way or two-way ANOVA with a post-hoc Holm-Sidak test. Here, ns, not significant; ∗p < 0.05; ∗∗p < 0.01; ∗∗∗p < 0.001; ∗∗∗∗p < 0.0001; compared with the control group.

Next, we assessed myocardial cytotoxicity over a period of 24 h. Following DOX treatment, apoptosis increased in the KO CMs (Figures 3A–3C) and decreased in the KI CMs (Figures S10A–S10C). Calcein/propidium iodide staining showed that, compared with controls, KO CMs had decreased survival (Figures 3D and 3E), while survival of KI CMs increased (Figures S10D and S10E). In summary, our findings demonstrate that ALKBH5 plays an important role in DOX-induced myocardial injury.

### ALKBH5 regulates DOX-induced mitochondrial dysfunction

**Figure 4:**
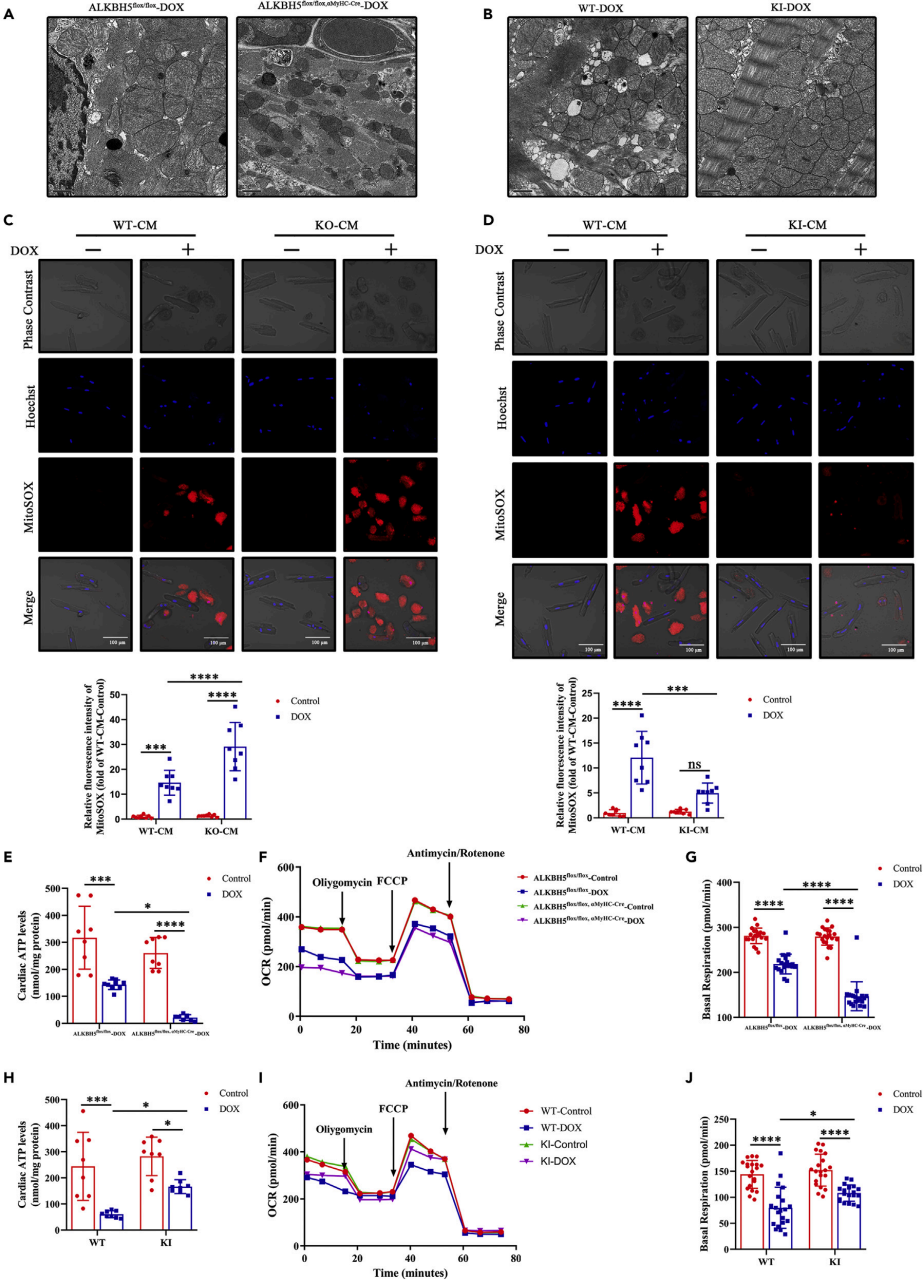
ALKBH5 regulates DOX-induced mitochondrial dysfunction (A) Representative images of mitochondrial morphology in myocardial tissue with or without DOX in ALKBH5^flox/flox^ and ALKBH5^flox/flox, α−MyHC-Cre^ mice (Bar = 1 μm). (B) Representative images of mitochondrial morphology in myocardial tissue with or without DOX in WT-DOX and KI-DOX mice (Bar = 1 μm). (C) Representative images and analysis of mitochondrial ROS level changes (MitoSOX red fluorescence) resulting from DOX treatment with or without DOX treatment in in ALKBH5-KO-CM and WT-CM (Bar = 100 μm, n > 6). (D) Representative images and analysis of mitochondrial ROS level changes (MitoSOX red fluorescence) resulting from DOX treatment with or without DOX treatment in ALKBH5-KI-CM and WT-CM (Bar = 100 μm, n > 6). (E) ATP production of myocardial tissue with or without DOX treatment in ALKBH5^flox/flox^ and ALKBH5^flox/flox, α−MyHC-Cre^ mice (n > 6). (F) OCRs changes of cardiomyocytes with or without DOX treatment in ALKBH5^flox/flox^ and ALKBH5^flox/flox, α−MyHC-Cre^ mice. (G) Statistical analysis of basal respiration with or without DOX treatment in ALKBH5^flox/flox^ and ALKBH5^flox/flox, α−MyHC-Cre^ mice (n > 6). (H) ATP production of myocardial tissue with or without DOX treatment in WT and KI mice (n > 6). (I) OCRs changes of cardiomyocytes with or without DOX treatment in WT and KI mice. (J) Statistical analysis of basal respiration with or without DOX treatment in WT and KI mice (n > 6). Data are depicted as the mean ± SEM. Statistical significance was determined by Student’s *t* test, one-way or two-way ANOVA with a post-hoc Holm-Sidak test. Here, ns, not significant; ∗p < 0.05; ∗∗p < 0.01; ∗∗∗p < 0.001; ∗∗∗∗p < 0.0001; compared with the control group.

We next evaluated ALKBH5 function in mitochondria. Electron microscopy showed that DOX caused disordered mitochondrial arrangement and larger mitochondria in *Alkbh5*^*flox/flox*, α−MyHC-Cre^ mice compared with control mice (Figure 4A), whereas *Alkbh5*-KI mice showed more intact mitochondrial cristae (Figure 4B). As the loss of mitochondrial membrane potential induces mitochondrial dysfunction leading to apoptosis, we extracted myocardial mitochondria and assessed JC-1 levels by flow cytometry. *Alkbh5*^*flox/flox*, α−MyHC-Cre^ mice showed lower mitochondrial membrane potential after DOX treatment (Figures S11A and S11C), whereas it was significantly increased by ALKBH5 overexpression (Figures S11B and S11D). Consistently, cellular experiments also showed that ALKBH5 significantly increased mitochondrial membrane potential (Figures S11E–S11H). Next, we assessed mitochondrial ROS using mitoSOX. Consequently, ALKBH5-deficient CMs showed significantly increased levels (Figure 4C), whereas ALKBH5-overexpressing CMs showed decreased mitochondrial ROS (Figure 4D).

Because mitochondrial dysfunction affects CM energetic homeostasis, we next measured myocardial ATP production in mice. It was inhibited in ALKBH5-deficient myocardium (Figure 4E) whereas ALKBH5 overexpression significantly promoted it (Figure 4H). Next, we assessed cellular basal respiration by oxygen consumption rates (OCRs). Interestingly, ALKBH5 deficiency decreased (Figures 4F and 4G), whereas ALKBH5 overexpression significantly increased myocardial basal respiration compared with controls (Figures 4I and 4J).

Because the mechanical regulation of CM contractility relies on a constant energy supply from mitochondria, we measured the effect of ALKBH5 on the contractility of individual CMs. Mitochondrial respiration-dependent contractility was significantly decreased in *Alkbh5*-KO (Figures S4F–S4K) and *Alkbh5*^*flox/flox*, α−MyHC-Cre^ mice (Figures S7B–S7G), while in *Alkbh5*-KI mice it was significantly enhanced after DOX treatment (Figures S5F–S5K). Taken together, our data indicate that ALKBH5 increases myocardial mitochondrial membrane potential, MitoSOX, ATP production, and basal respiration and maintains CM energetic homeostasis, protecting the myocardium from DOX-induced mitochondrial dysfunction.

### Identification of potential ALKBH5 target genes

**Figure 5:**
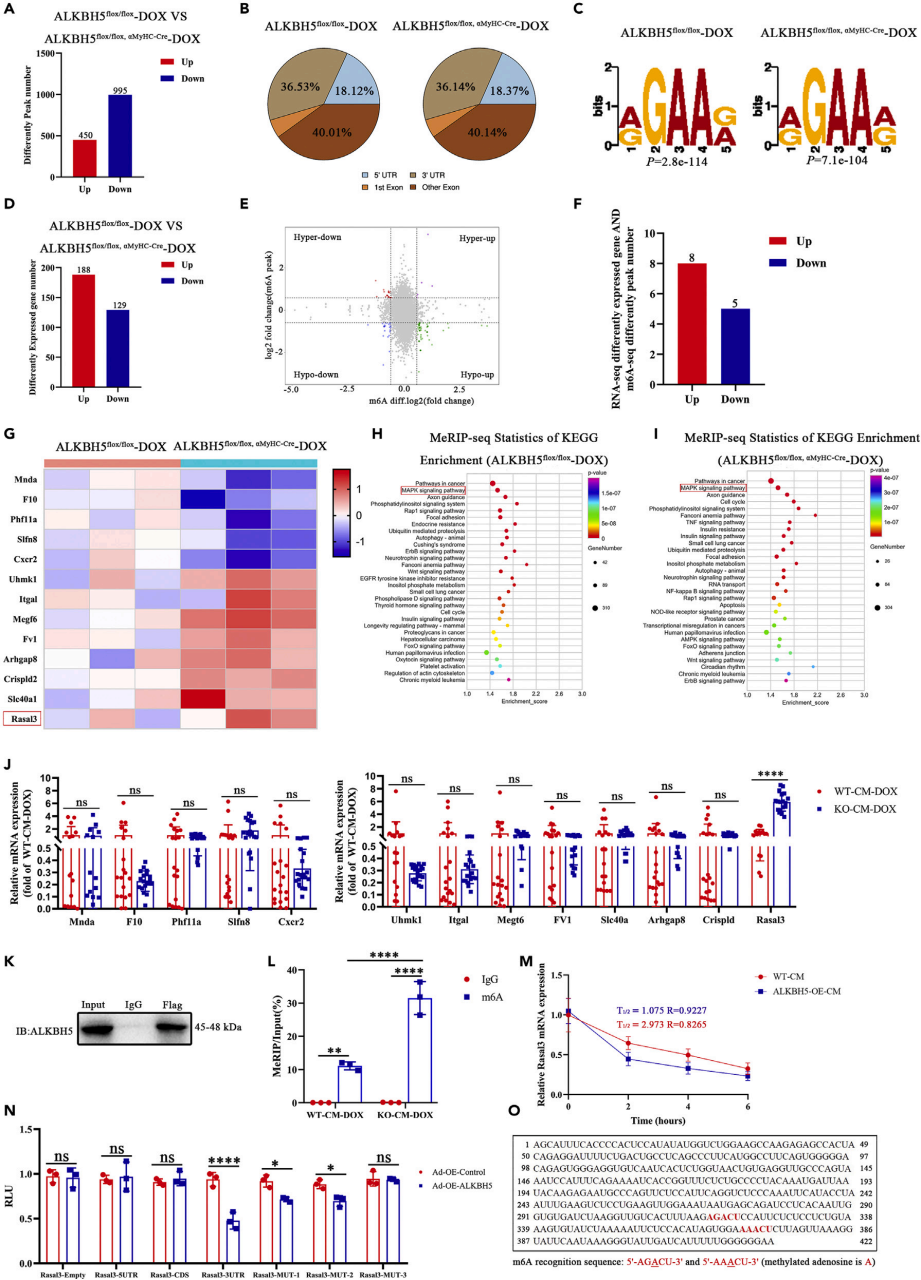
MeRIP-seq combined with RNA-seq revealed potential target genes of ALKBH5 (A) MeRIP-seq statistics showed the numbers of differentially methylated peaks between the ALKBH5^flox/flox^-DOX and ALKBH5^flox/flox, α−MyHC-Cre^-DOX groups. (B) Distribution of m6A peaks throughout mRNA lengths. (C) Representative motif analysis of ALKBH5^flox/flox^-DOX and ALKBH5^flox/flox, α−MyHC-Cre^-DOX. (D) RNA-seq showed the differentially expressed genes between the ALKBH5^flox/flox^-DOX and ALKBH5^flox/flox, α−MyHC-Cre^-DOX groups. (E) Scatterplot showing the distribution of the expression and m6A modification levels of genes (the first quadrant of the four quadrants represents differential genes with upregulated methylation and upregulated expression; the second quadrant represents differential genes with upregulated methylation and downregulated expression; the third quadrant represents the differential genes whose methylation is downregulated while their expression is downregulated; the fourth quadrant represents differential genes whose methylation was downregulated and their expression was upregulated). (F) Combined differential gene analysis of m6A-seq and RNA-seq revealed relevant regulatory genes (Bar chart showing the number of differential genes). (G) Heatmap showing differentially expressed abundance. (H) MeRIP-seq statistics of ALKBH5^flox/flox^-DOX group peak-related genes KEGG Enrichment. (I) MeRIP-seq statistics of ALKBH5^flox/flox, α−MyHC-Cre^-DOX group peak-related genes KEGG Enrichment. (J) RT-qPCR validation of Rasal3, Slc40a1, Crispld2, Arhgap8, Fv1, Megf6, Itgal, Uhmkl1, Cxcr2, Slfn8, Phf11a, F10, and Mnda expression after WT-CM-DOX and KO-CM-DOX transfection (n > 6). (K) Proteins were immunoprecipitated with either an FLAG or control IgG antibody. (L) RIP analysis of adult mouse cardiomyocyte products by RT-qPCR. (M) RT-qPCR showing the half-life of Rasal3-mRNA by monitoring the transcript abundance after transcriptional inhibition with actinomycin D at different time points in WT-CM or ALKBH5-OE-CM transfection (non-linear regression). (N) Dual-luciferase reporter assay after co-transfection with the reporter vector and ALKBH5-OE-Control or ALKBH5-OE in 293T cells for 48 h. (O) Rasal3-UTR sequence analysis displaying two m6A target sequences (5′-AGACU-3′ and 5′-AAACU-3′). Data are depicted as the mean ± SEM. Statistical significance was determined by Student’s *t* test or one-way ANOVA or two-way ANOVA with a post-hoc Holm-Sidak test. Here, ns, not significant; ∗p < 0.05; ∗∗p < 0.05; ∗∗∗p < 0.001; ∗∗∗∗p < 0.0001.

To address the mechanism by which ALKBH5 ameliorates DIC injury, we combined methylated RNA immunoprecipitation sequencing (MeRIP-seq) and RNA sequencing (RNA-seq) to *Alkbh5*^*flox/flox,*^ ^α−MyHC-Cre^ mice and *Alkbh5*^*flox/flox*^ control myocardia. MeRIP-seq identified 995 hypomethylated and 450 hypermethylated peaks (Figure 5A); the distribution of differentially methylated mRNA peaks is shown in Figure 5B. Analysis of representative motifs from DOX-treated myocardia was performed (Figure 5C). RNA-seq revealed 188 upregulated and 129 downregulated genes in *Alkbh5*^*flox/flox,*^ ^α−MyHC-Cre^ mice compared with controls (Figure 5D). Furthermore, m^6^A levels in the corresponding genes indicated that ALKBH5 affected the expression of multiple genes via m^6^A demethylation on day 7 of DOX treatment (Figure 5E). Combined analysis of the RNA-seq-identified differentially expressed genes (Figure 5F) and m^6^A-seq-based differential peak numbers showed that eight genes (*Rasal3, Slc40a1, Crispld2, Arhgap8, Fv1, Megf6, Itgal*, and *Uhmkl1*) were upregulated and five (*Cxcr2, Slfn8, Phf11a, F10*, and *Mnda*) were downregulated in *Alkbh5*^*flox/flox,*^ ^α−MyHC-Cre^ mice compared with *Alkbh5*^*flox/flox*^ controls (Figure 5G). Pathway analysis of MeRIP-seq peak-related genes using the Kyoto Encyclopedia of Genes and Genomes (KEGG) database revealed enrichment of the mitogen-activated protein kinase signaling pathway with the repair of DIC-caused damage (Figures 5H and 5I).

Based on the comparison of these transcripts with a subset of transcripts identified and on pathway analysis by MeRIP-seq, we hypothesized that Rasal3 is involved in DIC repair following ALKBH5 deficiency. To test this hypothesis, we extracted CMs from *Alkbh5*-KO and WT control mice and subjected them to DOX treatment. We found that *Rasal3* mRNA expression was significantly higher in KO CMs (Figure 5J). However, the other 12 genes (*Slc40a1, Crispld2, Arhgap8, Fv1, Megf6, Itgal, Uhmkl1, Cxcr2, Slfn8, Phf11a, F10*, and *Mnda*) did not show significant mRNA expression differences (Figure 5J). Because we were unable to obtain immunoprecipitation (IP)-grade ALKBH5 antibodies, we constructed an adenovirus-tagged ALKBH5 expression vector (Ad-ALKBH5-FLAG) and performed RNA-IP in transfected adult mouse CMs using anti-FLAG or immunoglobulin G (IgG) control antibodies to test for a direct interaction between ALKBH5 and *Rasal3* mRNA (Figures 5K and 5L). The anti-FLAG antibody increased precipitated *Rasal3* mRNA over that observed with the IgG control antibody (Figures 5K and 5L). Given that demethylation of m^6^A by ALKBH5 has been shown to affect mRNA stability,^23^ we extracted cardiomyocytes from adult ALKBH5-KI and control mice and measured Rasal3 mRNA levels 0, 2, 4, and 6 h after inhibiting RNA polymerase with actinomycin D. RT-qPCR revealed that ALKBH5 overexpression resulted in reduced half-life of Rasal3 mRNA compared with WT-CM group, suggesting that increased ALKBH5 destabilized Rasal3 mRNA (Figure 5M).

Next, we constructed *Rasal3* mRNA 5′-untranslated region (Rasal3-5′-UTR), coding sequence (Rasal3-CDS), Rasal3-3′-UTR, and control (Rasal3-empty) luciferase reporter plasmids to verify whether *Rasal3* mRNA is a direct target of ALKBH5. We found that ALKBH5 overexpression reduced luciferase activity from the Rasal3-3′-UTR plasmid but not that of the other plasmids (Figure 5N). We identified two m^6^A recognition sequences (5′-AGACU-3′ and 5′-AAACU-3′) within the 3′-UTR (Figure 4N). Next, the two m^6^A recognition sequences and three mutation sites were modified (5′-AGACU-3–5′-AGUCU-3′, Rasal3-MUT-1; 5′-AAACU-3–5′-AAUCU-3′, Rasal3-MUT-2; 5′-AGACU-3–5′-AGUCU-3′ and 5′-AAACU-3–5′-AAUCU-3′, Rasal3-MUT-3) and subsequently doubled (Figures 5N and 5O). The luciferase assay showed that ALKBH5 overexpression reduced the activity of the Rasal3-3′-UTR, -3′-UTR-1, and -3′-UTR-2 plasmids but had no effect on the other plasmids (Figures 5N and 5O).

To confirm whether ALKBH5 promotes CM apoptosis through m^6^A modification of *Rasal3* mRNA, AC16 cells were transfected with dCas13b-ALKBH5 and guide RNAs (gRNAs 1–3) targeting *Rasal3* (Figures S12A and S12B). RT-qPCR showed that the dCas13b-ALKBH5 combined with the gRNAs significantly decreased *Rasal3* mRNA expression compared with that with control non-targeting gRNA (NT-gRNA) (Figure S12C). dCas13b-ALKBH5 combined with gRNAs significantly decreased Rasal3 protein expression, cleaved caspase-3, and BAX compared with that with NT-gRNA (Figures S12D–S12G). In brief, these findings confirm that ALKBH5 regulates *Rasal3* mRNA expression.

### ALKBH5 exerts cardioprotective effects by promoting RAS/RAF/ERK signaling via m^6^A demethylation of Rasal3 mRNA

**Figure 6:**
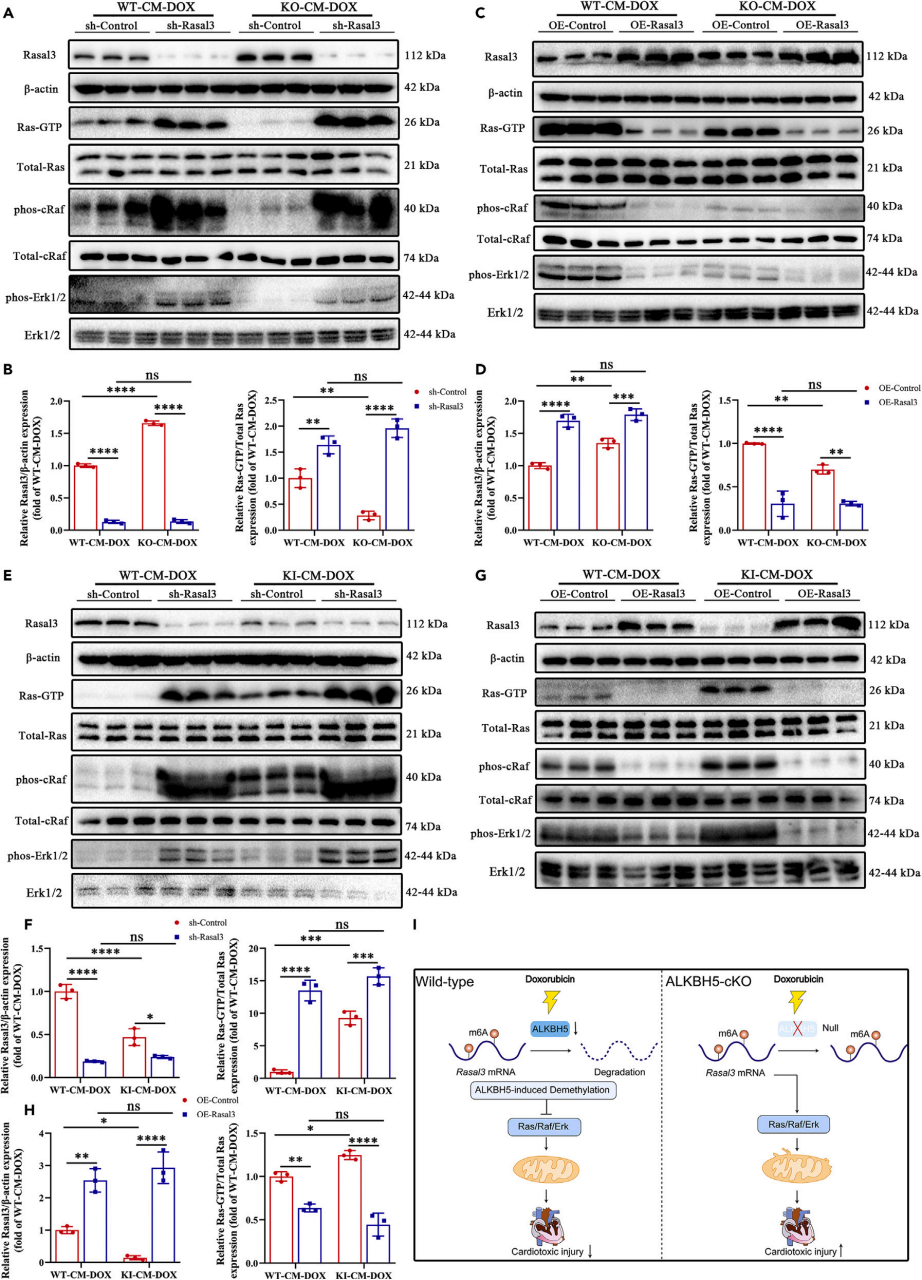
ALKBH5 exerts cardioprotective effects by promoting the Ras/Raf/Erk signaling pathway via m6A demethylation of Rasal3 mRNA (A) Representative western blots of Rasal3, Ras-GTP (active Ras), phosphorylated cRaf (phos-cRaf), and phosphorylated Erk (phos-Erk1/2) in ALKBH5-KO-CM-DOX and WT-CM-DOX after shRNA-Rasal3 knockdown. (B) Western blot analysis of Rasal3 and Ras-GTP in ALKBH5-KO-CM-DOX and WT-CM-DOX after shRNA-Rasal3 knockdown (n = 3). (C) Representative western blots of Rasal3, Ras-GTP (active Ras), phosphorylated cRaf (phos-cRaf), and phosphorylated Erk (phos-Erk1/2) in ALKBH5-KO-CM-DOX and WT-CM-DOX after OE-Rasal3 overexpression. (D) Western blot analysis of Rasal3 and Ras-GTP in ALKBH5-KO-CM-DOX and WT-CM-DOX after shRNA-Rasal3 knockdown (n = 3). (E) Representative western blots of Rasal3, Ras-GTP (active Ras), phosphorylated cRaf (phos-cRaf), and phosphorylated Erk (phos-Erk1/2) in ALKBH5-KI-CM-DOX and WT-CM-DOX after shRNA-Rasal3 knockdown. (F) Western blot analysis of Rasal3 and Ras-GTP in ALKBH5-KI-CM-DOX and WT-CM-DOX after shRNA-Rasal3 knockdown (n = 3). (G) Representative western blots of Rasal3, Ras-GTP (active Ras), phosphorylated cRaf (phos-cRaf), and phosphorylated Erk (phos-Erk1/2) in ALKBH5-KI-CM-DOX and WT-CM-DOX after OE-Rasal3 overexpression. (H) Western blot analysis of Rasal3 and Ras-GTP in ALKBH5-KI-CM-DOX and WT-CM-DOX after shRNA-Rasal3 knockdown (n = 3). (I) A proposed model showing how ALKBH5 mediates mitochondrial dysfunction to induce CM death and mitigate DIC injury. Data are depicted as the mean ± SEM. Statistical significance was determined by Student’s *t* test or one-way ANOVA or two-way ANOVA with a post-hoc Holm-Sidak test. Here, ns, not significant; ∗p < 0.05; ∗∗p < 0.05; ∗∗∗p < 0.001; ∗∗∗∗p < 0.0001.

To assess RAS/RAF/ERK signaling, we cultured CMs from adult *Alkbh5*-KO, *Alkbh5*-KI, and WT control mice and then measured the expression of proteins of the pathway. In KO CMs, Rasal3 expression was increased and accompanied by substantial decreases in Ras-GTP (active Ras), phosphorylated cRAF, and phosphorylated ERK levels (Figures 6A–6D and S13A–S13D). Interestingly, ALKBH5 overexpression inhibited Rasal3 and further increased Ras-GTP (activated Ras), phosphorylated cRAF, and phosphorylated ERK levels (Figures 6E–6H and S13E–S13H).

We next knocked down and overexpressed *Rasal3*. We found that RAS/RAF/ERK signaling was significantly activated by *Rasal3* knockdown in both KO and KI CMs but was significantly inhibited by *Rasal3* overexpression (Figures 6A–6H and S13A–S13H). Even more surprisingly, after *Rasal3* knockdown and overexpression, the effect of ALKBH5 on the activation of RAS/RAF/ERK signaling in the DIC model was significantly attenuated (Figures 6A–6H, and S13A–S13H). Taken together, these data strongly support the protective effect of ALKBH5 on the activation of RAS/RAF/ERK signaling by mediating *Rasal3* m^6^A methylation (Figure 6I).

### Knockdown of Rasal3 can antagonize the effect of ALKBH5 and alleviate DIC injury

**Figure 7:**
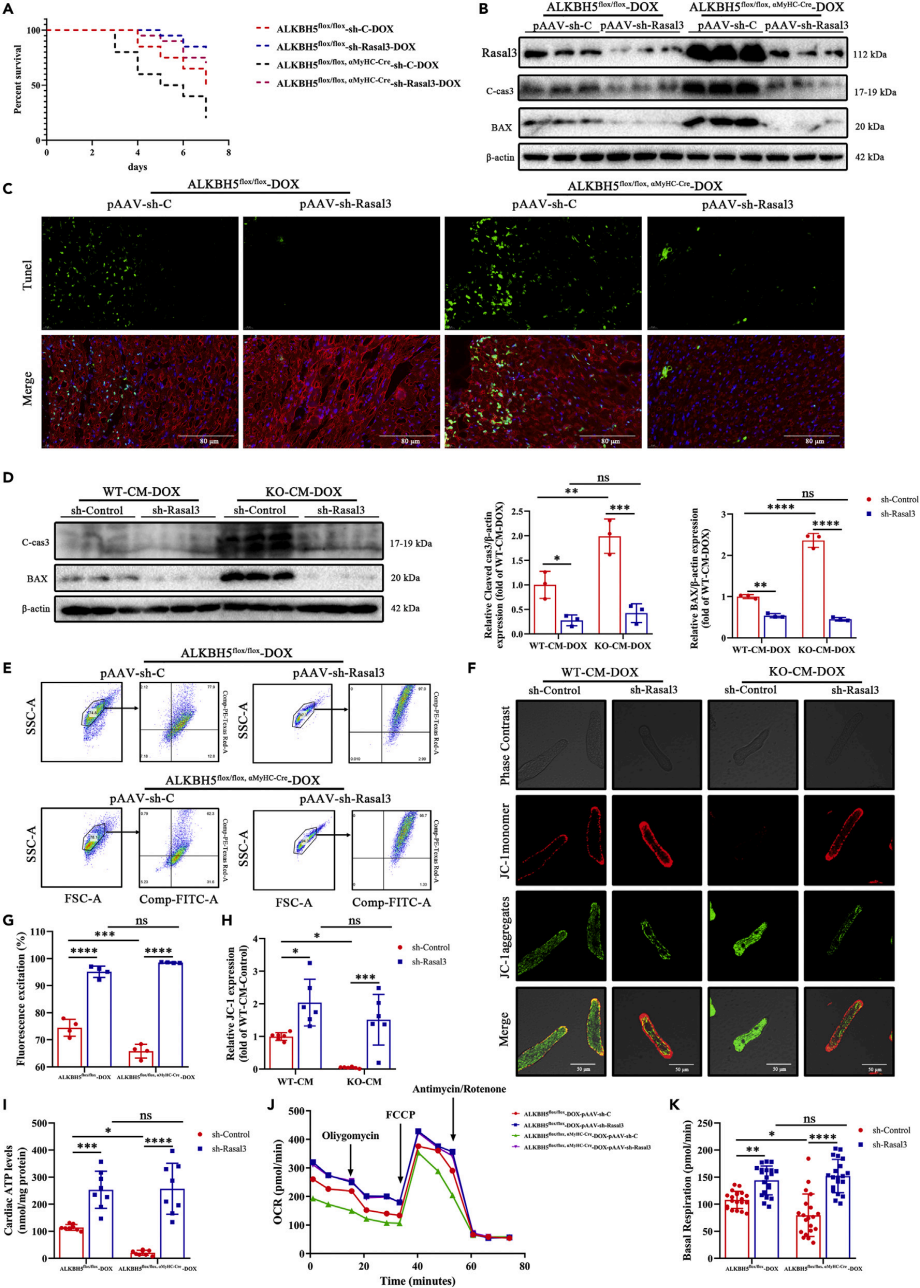
Adenovirus knockdown of Rasal3 can antagonize the effect of ALKBH5 and alleviate myocardial DIC injury (A) Kaplan-Meier survival curves showing the survival of DOX-stressed mice in ALKBH5^flox/flox^ and ALKBH5^flox/flox, α−MyHC-Cre^ after pAAV-shRNA-Rasal3 knockdown (n = 20). (B) Representative western blots of Rasal3, cleaved caspase-3, and BAX in ALKBH5^flox/flox^ and ALKBH5^flox/flox, α−MyHC-Cre^ after pAAV-shRNA-Rasal3 knockdown (n = 3). (C) Apoptosis measured by TUNEL staining in ALKBH5^flox/flox^ and ALKBH5^flox/flox, α−MyHC-Cre^ heart sections (Bar = 80 μm). (D) Western blot analysis of cleaved caspase-3 and BAX in ALKBH5-KO-CM-DOX and WT-CM-DOX after shRNA-Rasal3 knockdown (n = 3). (E and G) Flow cytometric detection of mitochondrial membrane potential JC-1 in myocardial tissue of with or without adenovirus knockdown of Rasal3 in ALKBH5^flox/flox^ and ALKBH5^flox/flox, α−MyHC-Cre^ mice (n = 4). (F and H) JC-1 changes resulting from shRNA-Rasal3 knockdown in ALKBH5-KO-CM and WT-CM (n = 6). (I) ATP production of myocardial tissue with or without DOX treatment in ALKBH5^flox/flox^ and ALKBH5^flox/flox, α−MyHC-Cre^ mice (n > 6). (J) OCRs changes of cardiomyocytes with or without adenovirus knockdown of Rasal3 in ALKBH5^flox/flox^ and ALKBH5^flox/flox, α−MyHC-Cre^ mice. (K) Statistical analysis of basal respiration with or without adenovirus knockdown of Rasal3 in ALKBH5^flox/flox^ and ALKBH5^flox/flox, α−MyHC-Cre^ mice (n > 6). Data are depicted as the mean ± SEM. Statistical significance was determined by Student’s *t* test or one-way ANOVA or two-way ANOVA with a post-hoc Holm-Sidak test. Here, ns, not significant; ∗p < 0.05; ∗∗p < 0.05; ∗∗∗p < 0.001; ∗∗∗∗p < 0.0001.

We next used adeno-associated virus pAAV-cTNT-miR30 short hairpin RNA to knock down *Rasal3* expression in *Alkbh5*^*flox/flox,*^ ^α−MyHC-Cre^ and *Alkbh5*-KI mice. *Rasal3* knockdown increased the survival of both control *Alkbh5*^*flox/flox*^ (80% vs 50%; log rank test; p = 0.0426; Figure 7A) and *Alkbh5*^*flox/flox,*^ ^α−MyHC-Cre^ (70% versus 20%; log rank test; p = 0.0009; Figure 7A) mice over that of controls. An apoptotic protein assay and TUNEL staining showed that *Rasal3* knockdown significantly attenuated myocardial apoptosis in both *A*LKBH*5*^*flox/flox*^ and *Alkbh5*^*flox/flox,*^ ^α−MyHC-Cre^ mice (Figures 7B, 7C, and S14A–S14D). The corresponding CM experiments confirmed that, after *Rasal3* knockdown, CM apoptosis was significantly reduced (Figure 7D).

Similar findings were obtained in *Alkbh5*-KI and control mice. Compared with that of DOX-treated *Alkbh5*-KI mice, the survival of *Alkbh5*-KI mice was significantly improved by *Rasal3* knockdown (65% vs 90%; log rank test; p = 0.0489; Figure S15A). *Rasal3* knockdown reduced myocardial apoptosis in both KI and WT mice (Figures S15B–S15G). The CM experiment further confirmed the above findings (Figure S15H).

To our surprise, the increased mortality of DIC mice caused by ALKBH5 deficiency was not only significantly reversed by *Rasal3* knockdown but also the survival was not significantly different from that of *Alkbh5*^*flox/flox*^ controls (70% vs 80%, respectively; log rank test; p = 0.5735; Figure 7A). This phenomenon was also observed in KI mice. Moreover, the protective effect of ALKBH5 overexpression on the survival rate of DIC mice after *Rasal3* knockdown was not significantly different from that in WT mice (90% vs 85%, respectively; log rank test; p = 0.6104; Figure S15A). The effects of ALKBH5 deficiency and overexpression on CM apoptosis in DIC mice after Rasal3 knockdown were both attenuated (Figures 7B–7D and S15B–S15H). Therefore, we conclude that the knockdown of *Rasal3* antagonizes the effect of ALKBH5 to alleviate DIC injury.

Next, we found that *Rasal3* knockdown significantly enhanced mitochondrial membrane potential (Figures 7E and 7G). Consistently, cellular experiments also showed similar results (Figures 7F and 7H). Interestingly, we found that *Rasal3* knockdown not only enhanced CM mitochondrial membrane potential but also showed no significant difference between *Alkbh5*-KO and WT control mice (Figures 7E–7H). Furthermore, we found that *Rasal3* knockdown promoted ATP production and improved basal respiration in both *Alkbh5*^*flox/flox,*^ ^α−MyHC-Cre^ and *Alkbh5*^*flox/flox*^ mice (Figures 7J and 7K). Similar to the trend of mitochondrial membrane potential, ATP production and basal respiration were not significantly different between *Alkbh5*^*flox/flox,*^ ^α−MyHC-Cre^ and *Alkbh5*^*flox/flox*^ mice after *Rasal3* knockdown (Figures 7I–7K).

### Rasal3 overexpression aggravates DIC injury

We next used the H20133 pAAV-cTNT-MCS-3×FLAG-tWPA vector to construct the Rasal3-overexpressing adenovirus pAAV-cTNT-OE-Rasal3 for *Alkbh5*^*flox/flox,*^ ^α−MyHC-Cre^ and *Alkbh5*-KI mice to overexpress myocardial Rasal3. We found that overexpression of Rasal3 reduced ALKBH5^flox/flox^ (55% vs 20%; log rank test; p = 0.0289; Figure S16A) and increased cardiomyocyte apoptosis compared with *ALKBH5*^*flox/flox*^-OE controls (Figures S16B–S16H). However, compared with the *Alkbh5*^*flox/flox,*^ ^α−MyHC-Cre^-OE controls, after overexpression of Rasal3 in the *Alkbh5*^*flox/flox,*^ ^α−MyHC-Cre^ myocardium, there was no significant difference in survival of DIC mice (30% vs 15%; log rank test; p = 0.6447; Figure S16A), but overexpression of Rasal3 led to a significant increase in myocardial apoptosis in DIC mice (Figures S16B–S16H). In addition, compared with the *Alkbh5*-KI-OE controls, the overexpression of Rasal3 significantly reduced the survival of DIC *Alkbh5*-KI mice (50% versus 10%; log rank test; p = 0.0397; Figure S17A) and significantly increased CM apoptosis (Figures S17B–S17H).

Consistent with the results shown in Figure 6, there was no significant difference in survival between *Alkbh5*^*flox/flox*^ control and *Alkbh5*^*flox/flox,*^ ^α−MyHC-Cre^ mice (20% versus 15%, respectively; log rank test; p = 0.7271; Figure S17A) or in myocardial apoptosis after Rasal3 overexpression (Figures S16B–S16H). In addition, we found that both survival (10% versus 30%; log rank test; p = 0.2951; Figure S17A) and myocardial apoptosis (Figures S17B–S17H) in *Alkbh5*-KI mice after Rasal3 overexpression were not significantly different from those in WT mice. In short, our results demonstrate that Rasal3 overexpression attenuates the protective effects of *Alkbh5* deletion and overexpression in DIC mice.

## Discussion

The findings of our study demonstrate that mitochondrial dysfunction and CM apoptosis due to DIC can be reversed by ALKBH5-mediated m^6^A demethylation. The acute DIC and CDIC model data from mice with three genotypes, as well as data from mice after adenovirus intervention, support the conclusion that ALKBH5-targeted interventions have therapeutic potential for DIC. Our data further indicate that ALKBH5 deficiency promotes mitochondrial damage and CM apoptosis. In addition, ALKBH5 affects *Rasal3* mRNA stability through m^6^A demethylation and activates RAS, thus inhibiting CM apoptosis through the RAS/RAF/ERK signaling pathway and ultimately attenuating acute DIC injury.

The m6A base modification is common in mRNA, and such modifications often maintain mRNA stability.^24^^,^^25^^,^^26^ The methylation of m^6^A is reversible, and its regulatory factors include methyltransferases, demethylases, and methylation-reading proteins, which play important roles in the occurrence and progression of cardiovascular disease.^26^^,^^27^ However, the role of the m^6^A demethylase ALKBH5 in DIC has not been previously reported. Our results fill this gap and reveal that ALKBH5-mediated m^6^A demethylation is a key driver in the regulation of the repair of DIC-caused damage.

We found that the levels of myocardial m^6^A were significantly increased while the expression of ALKBH5 was significantly downregulated in mice with DIC, suggesting a role for ALKBH5. Therefore, we constructed *Alkbh5*-KO and *Alkbh5*-KI mice and found that the *Alkbh5*-KO mice exhibited higher mortality and aggravated DIC injury. Cardiotoxicity was alleviated in the *Alkbh5*-KI mice, and their survival increased, suggesting that ALKBH5 overexpression protects the myocardium from DIC injury.

Cardiac tissue is mainly composed of CMs, endothelial cells, fibroblasts, and immune cells, such as macrophages and lymphocytes, with CMs playing an important role in DIC.^1^^,^^28^^,^^29^ To determine whether ALKBH5 plays a role in regulating m^6^A methylation in CMs, we constructed a murine DIC model with CM-specific deletion of *Alkbh5* and observed mitochondrial damage, including dissipation of the mitochondrial membrane potential, uncoupling of ATP depletion, ROS production, and increased myocardial apoptosis and thus significant deterioration of myocardial function. *In vitro* and *in vivo* experimental results in adult mouse CMs were consistent, providing evidence that the Alkbh5 deletion in CMs further aggravates DIC.

To explore the mechanism by which ALKBH5 improves mitochondrial function and regulates myocardial injury repair in DIC, we combined differentially expressed transcripts from RNA-seq data and differentially methylated transcripts from MeRIP-seq data to screen for 12 differential genes. In addition, we combined the KEGG pathway analysis of MeRIP-seq peak-related genes and molecular experiments to confirm that Rasal3 was a target gene regulated by ALKBH5 for DIC damage repair. Therefore, we speculate that m^6^A demethylation by ALKBH5 may mediate Rasal3 mRNA stability to regulate DIC damage repair. RAS, a member of the GTPase family, is a small monomeric GTP-binding protein composed of 190 amino acid residues.^30^^,^^31^^,^^32^^,^^33^ It has GTPase activity and is located on the cytoplasmic side of the plasma membrane.^34^^,^^35^^,^^36^ The RAS protein binds to the N-terminal domain of RAF and activates the RAS/RAF/ERK pathway.^37^^,^^38^ ERK1/2, which are important signaling molecules in the RAS pathway, are located downstream of RAS.^39^^,^^40^^,^^41^ As an RAS inhibitor, RASAL3 can inhibit RAS activity, thereby regulating the RAS signaling pathway.^42^^,^^43^^,^^44^ Our study showed that Rasal3 expression was significantly upregulated following ALKBH5 deletion. We further found that ALKBH5 overexpression reduced the stability of Rasal3 mRNA following actinomycin D treatment. Dual-luciferase reporter and RIP assays demonstrated that ALKBH5 could bind to and demethylate Rasal3 mRNA at two 3′-UTR m^6^A residues, thus destroying the stability of the transcripts. These findings support the hypothesis that ALKBH5-mediated m^6^A demethylation can lead to the downregulation of Rasal3 expression.

We demonstrated in both *in vivo* and *in vitro* rescue experiments that knockdown of *Rasal3* in CMs reduced CM apoptosis and mortality in DIC mice through the RAS/RAF/ERK pathway. Furthermore, it was even more surprising that *Rasal3* knockdown in CMs reversed DIC injury in mice that was caused by *Alkbh5* deletion, while overexpression of Rasal3 antagonized the cardioprotective effects of ALKBH5 overexpression. In conclusion, this study reveals a novel link between DIC and the m^6^A demethylase ALKBH5. Demethylation of m^6^A by ALKBH5 in CMs affects the stability of *Rasal3* mRNA, leading to the activation of Ras, activation of the RAS/RAF/ERK pathway, reduction of myocardial apoptosis and ROS damage, and ultimate alleviation of DIC.

Mitochondria are important organelles of CM, providing approximately 90% of ATP production in cardiomyocytes.^45^^,^^46^ Especially under pathological conditions, the function of mitochondria is crucial.^2^^,^^8^^,^^30^^,^^46^^,^^47^ We found that after ALKBH5 deficiency, DOX induced mitochondrial dysfunction, inhibited ATP production and basal metabolic respiration, and aggravated myocardial injury. This is closely related to the inhibition of RAS/RAF/ERK activation by ALKBH5 deficiency. Numerous previous studies have indicated that activation of ERK1/2 improves mitochondrial function and inhibition of the RAS/RAF/ERK pathway leads to cell death.^48^ In response to toxic stress, inhibition of ERK1/2 and PD98059 promotes the release of cytochrome *c* from mitochondria into the cytoplasm, which in turn induces neuronal cell death.^48^^,^^49^ In contrast, blockade of CB2 receptors was protective in myocardial ischemia/reperfusion and increased ERK1/2 phosphorylation associated with decreased cytochrome *c* release and low PTP opening.^48^^,^^50^ Furthermore, in alveolar macrophages, RAS/RAF/ERK pathway has been shown to regulate mitochondrial integrity and ATP production, whereas ERK inhibition results in cell death.^48^^,^^51^ Our results further demonstrate that ALKBH5 protects mitochondrial function through RAS/RAF/ERK pathway to alleviate DIC injury.

In conclusion, our study clearly demonstrates the protective role of ALKBH5 in myocardial mitochondrial function and CM survival during DIC injury. ALKBH5-mediated *Rasal3* m^6^A demethylation activates RAS/RAF/ERK signaling to alleviate DIC injury. These novel findings facilitate the search for potential intervention targets to reduce the myocardial toxicity of anthracyclines in patients with cancer.

### Limitations of the study

Given the lack of previous studies on the roles of ALKBH5 and Rasal3 in DIC, this “proof-of-principle” study has certain limitations. We monitored the effect of ALKBH5 deficiency on the repair of cardiotoxic injury in both DIC and CDIC animal models, explored rescue strategies, and proposed new clinical treatment strategies. However, we only relied on animal models without further clinical patient data validation. Although the protective effects of overexpression of ALKBH5 and inhibition of Rasal3 on myocardial mitochondrial function and cardiomyocytes in DOX-treated mice are encouraging, further studies are needed to achieve early clinical translation.

## STAR★Methods

### Key resources table

**Table:** 

REAGENT or RESOURCE	SOURCE	IDENTIFIER
**Antibodies**		

Mouse monoclonal to Cardiac Troponin T	Abcam	Cat#ab8295
N6-Methyladenosine (m6A) Rabbit mAb	CST	Cat#56593
Rabbit monoclonal to ALKBH5	Abcam	Cat#ab195377
Rabbit monoclonal to METTL3	Abcam	Cat#ab195352
Rabbit polyclonal to METTL14	Abcam	Cat#ab252562
Rabbit monoclonal to FTO	Abcam	Cat#ab280081
Mouse monoclonal to Bax	Abcam	Cat#ab3191
Rabbit polyclonal to Bcl-2	Abcam	Cat#ab196495
Rabbit monoclonal to Cleaved Caspase-3	Abcam	Cat#ab214430
Raf1 Rabbit pAb	Abclone	Cat#A0223
Phospho-Raf1-S259 Rabbit mAb	Abclone	Cat#AP1012
Phospho-p44/42 MAPK (Erk1/2) (Thr202/Tyr204) (D13.14.4E) XP® Rabbit mAb	CST	Cat#4370S
β-actin (13E5) Rabbit mAb	CST	Cat#4970S
Rabbit Polyclonal to RASAL3	Novusbio	Cat#NBP2-83439
Goat anti-Mouse IgG (H + L) Alexa Fluor® Plus 488	Thermo	Cat#A32723

**Bacterial and virus strains**		

pAAV-cTNT-Rasal3-3xFLAG-tWPA	Obio	N/A
pAAV-cTNT-P2A-3xFLAG-miR30-shRNA (Rasal3)-WPRE	Obio	N/A
ALKBH5 adenoviral vectors and the corresponding negative controls	Obio	N/A
Rasal3 adenoviral vectors and the corresponding negative control	Obio	N/A

**Chemicals, peptides, and recombinant proteins**		

Doxorubicin	Sigma-Aldrich	Cat#25316-40-9
WGA dyes	Sigma-Aldrich	Cat#L4895
OCT embedding medium	Sakura	Cat#4583
PBS	Hyclone	Cat#BSS-PBS-1X6
DAPI	Solarbio	Cat#C0065
ROS staining solution	Beyotime	Cat#S0033
TRIzol reagent	Invitrogen	Cat#15,596,026
Hybond-N+ membrane	GE Healthcare	Cat#RPN203B
Actinomycin D	Sigma-Aldrich	Cat#50-76-0
Lipofectamine Reagent 3000	Invitrogen	Cat#L3000-015
NaCl	1^st^ Base	Cat#BIO-1111
KCl	Sigma-Aldrich	Cat#P9541
NaH2PO4	Sigma-Aldrich	Cat#S8282
HEPES	1^st^ Base	Cat#BIO-1825
Glucose	Sigma-Aldrich	Cat#G8270
BDM	Sigma-Aldrich	Cat#B0753
Taurine	Sigma-Aldrich	Cat#T8691
EDTA	Sigma-Aldrich	Cat#EDS
MgCl_2_	Sigma-Aldrich	Cat#M8266
Collagenase II	Worthington	Cat#LS004176
Collagenase IV	Worthington	Cat#LS004188
Protease XIV	Sigma-Aldrich	Cat#P5147
FBS	Thermo Scientific	Cat#10270106
Laminin	Thermo Scientific	Cat#23017-15
M199 Medium	Sigma-Aldrich	Cat#M4530
DMEM/F12 Medium	Thermo Scientific	Cat#11320-033
BSA	Sigma-Aldrich	Cat#A1470
ITS supplement	Sigma-Aldrich	Cat#I3146
Chemically defined lipid concentrate	Thermo Scientific	Cat#11905-031
Penicillin-Streptomycin	Thermo Scientific	Cat#15070-063

**Critical commercial assays**		

TUNEL staining kit	Roche	Cat#11684817910
CK-MB Enzyme-Linked Immunosorbent Assay kits	Sigma	Cat#MAK116
cTnT Enzyme-Linked Immunosorbent Assay kits	Novus Biologicals	Cat#MAB18742-100
Tissue mitochondrial isolation kit	Beyotime Biotechnology	Cat#C3606
Enhanced mitochondrial membrane potential assay kit with JC-1	Beyotime Biotechnology	Cat#C2003S
Enhanced ATP assay kit	Beyotime Biotechnology	Cat#S0027
The Prime-Script RT kit	TaKaRa	Cat#RR036A
Calcein AM/PI detection working solution	Beyotime	Cat#C2015L
Ras Activation Assay Kit	Bioyears	Cat#81101

**Deposited data**		

Raw and analysis data	This paper	GEO: GSE224215

**Experimental models: Cell lines**		

293T cell	Abiowell	Cat#AW-CNH086
AC16 cell	Abiowell	Cat#AW-CNH103

**Experimental models: Organisms/strains**		

C57/B6 mice	Shanghai Laboratory Animal Research Center	N/A
ALKBH5 (flox/flox) and ALKBH5(flox/flox, αMyHC-Cre)	Cyagen	N/A
ALKBH5-knockout (ALKBH5-KO) mice	Cyagen	N/A
ALKBH5-knockin (ALKBH5-KI) mice	Cyagen	N/A

**Software and algorithms**		

GraphPad Prism 8.0	Graphpad Software	https://www.graphpad.com/demos/
ImageJ	National Institutes of Health	https://imagej.en.softonic.com/

**Oligonucleotides**		

Primers for Figure 1, see Table S1	This paper	N/A
Primers for Figure 5, see Table S2	This paper	N/A
gRNA for Figure 5, see Table S3	This paper	N/A

### Resource availability

#### Lead contact

Further information and requests for resources and reagents should be directed to and will be fulfilled by the lead contact, Dr. Yi-Qing Yang (yangyiqing@fudan.edu.cn).

#### Materials availability

Available through lead contact.

### Experimental model and subject details

6–8-week-old male C57/B6 mice were maintained on a 12 h light/dark cycle. Mice were purchased from the Shanghai Laboratory Animal Research Center (Shanghai, China). All protocols were approved by the Animal Care Ethics Committee of Fudan University (No. 2021-034) and performed in accordance with the National Institutes of Health Guide for the Care and Use of Laboratory Animals. ALKBH5-knockout (ALKBH5-KO) and ALKBH5-knockin (ALKBH5-KI) mice were purchased from Gem Pharmatech (Nanjing, China). The specific design and identification results can be found in Expanded material 1 and Expanded material 2. ALKBH5 (^flox/flox^) and ALKBH5-myocardial specific knockout (ALKBH5^flox/flox, αMyHC−Cre^) mice were purchased from Cyagen (Suzhou, China). The protocol for establishing the doxorubicin-induced cardiotoxicity (DIC) model was based on a previous study.^52^^,^^53^^,^^54^ Intraperitoneal injections (IP) of DOX (20 mg/kg; Sigma, 25,316-40-9) were administered to the 6–8-week-old mice to construct mouse acute DIC model. Details can be found in Figure S1A. Mice were intraperitoneally injected with doxorubicin (5 mg/kg) or normal saline (NS) once a week for four weeks to construct mouse chronic DIC model. Details can be found in Figure S1B. All animal experiments were performed under specific sterile barrier conditions in accordance with institutional guidelines, and the experimental protocols were approved by the Ethics Committee of Animal Experimentation of Fudan University. For analgesia, carprofen (5 mg/kg) was administered subcutaneously at the time of intraperitoneal injections of DOX and every 24 h thereafter for 2 days. An additional dose of the analgesic was administered if the animals appeared to experience pain (based on criteria such as immobility and failure to eat). At the indicated time points, the mice were sacrificed by cervical dislocation under CO_2_ anesthesia, and tissues were harvested for analyses.

### Method details

#### Echocardiography analysis

Mice were anesthetized with isoflurane and cardiac function was evaluated. MI/RI and M-mode images were acquired using a Vevo 2100 high-frequency ultrasound system (VisualSonics, Toronto, ON, Canada). Data were averaged based on measurements of at least six cardiac cycles, including heart rate (BPM), left ventricular ejection fraction (LVEF), and left ventricular fractional shortening (LVFS) scores. The procedures were performed as previously described.^55^^,^^56^

#### Adenovirus-associated virus-transfected mice

Use pAAV-cTNT-MCS-3xFLAG-tWPA vector to construct Rasal3 overexpressing adeno-associated virus (pAAV-cTNT-Rasal3-3xFLAG-tWPA) purchased from Obio (Shanghai, China). Use pAAV-cTNT-P2A-3xFLAG-WPRE vector to construct Rasal3 knockdown adeno-associated virus (pAAV-cTNT-P2A-3xFLAG-miR30-shRNA (Rasal3)-WPRE) purchased from Obio (Shanghai, China). Adenovirus-associated virus infection was performed according to the recommended protocol. Briefly, 100 μL of 10E12v.g adeno-associated and control viruses were injected into the tail vein, and the mice were intervened after 4 weeks.

#### Adenoviral transduction cells

ALKBH5 adenoviral vectors (Ad-ALKBH5, Ad-Sh-ALKBH5) and the corresponding negative controls (Ad-Control, Ad-Sh-Control), Rasal3 adenoviral vectors (Ad-cTNT-Rasal3, Ad-Sh-Rasal3) and the corresponding negative control (Ad-cTNT-Control, Ad-Sh-Rasal3-Control) were purchased from Obio (Shanghai, China). Adenoviral infection was performed according to the recommended protocol. Briefly, 1 × 10^8^ pfu/mL of control and overexpression adenovirus were quantified and diluted in serum-free DMEM. Subsequently, the adenovirus mixtures were added to the cultured plates containing cardiomyocytes. The supernatants were discarded after 12 h and replaced with cardiomyocyte culture medium.

#### Systolic and diastolic cardiomyocyte function

Cardiomyocytes were isolated from the mice using a previously published method.^57^ The systolic and diastolic functions of primary cardiomyocytes were detected using the IonOptix™ system. The detection buffer, cardiomyocyte calcium buffer, which comprises 130 mM NaCl, 5.4 mM KCl, 10 mM HEPES, 1.8 mM CaCl2, 0.5 mM MgCl2, and 10 mM glucose, pH 7.4. Two drops of the buffer were added to each slide. The systolic and diastolic cardiomyocyte functions were assessed by measuring the following indicators: resting cell length, peak shortening (PS), time-to-PS (TPS), the maximal velocity of shortening (-dL/dt), the maximal velocity of re-lengthening (+dL/dt), and time-to-90% re-lengthening (TR90).

#### Histological analysis

Myocardial tissue from the DIC mouse model was fixed using 4% paraformaldehyde and then embedded and fixed in paraffin. Paraffin sections were dewaxed as follows. The paraffin section was placed in xylene-I for 20 min, followed by xylene-II for 20 min, absolute ethanol-I for 5 min, absolute ethanol-II for 5 min, 75% alcohol for 5 min, and then washed with water. The sections were then stained following antigen repair. WGA (L4895, Sigma) dyes were used for staining. Detailed methods have been described previously.^58^^,^^59^

#### TUNEL assay

Myocardial tissue was wrapped with OCT embedding medium (4583, Sakura) and 2–3 μm tissue sections cut using a microtome. Each section was dried slightly, a circle was drawn around the tissue with a histochemical pen (to prevent the liquid from flowing away), protease K (Biofroxx) working solution was dropped in the circle to cover the tissue, and the tissue was incubated in a 37 °C incubator for 25 min. The slide was placed in PBS (pH7.4, Hyclone), shaken, and washed three times on the decolorization shaking table, each time for 5 min. After the tissue sections were broken, they were washed three times with PBS. The samples were incubated with TUNEL staining solution (TUNEL staining kit, 11684817910, Roche) for 2 h at 37 °C. After blocking with PBS, samples were incubated overnight at 4 °C with the primary antibody (anti-cardiac troponin T; ab8295, Abcam) and then with the secondary antibody for 2 h at 37 °C (Alexa Fluor Plus 488; A32723, Thermo). DAPI (C0065, Solarbio) was used to counterstain the nuclei, which were then photographed.

#### Measurement of reactive oxygen species (ROS)

Myocardial tissue was fixed and sectioned as previously mentioned. ROS staining solution (S0033; Beyotime) was used for staining. The nuclei were counterstained with DAPI and photographed.

#### Mitochondrial ROS analysis

The detection of Mitochondrial ROS was as described previously.^55^ In short, MitoSOX Red Superoxide Indicator (Invitrogen, Carlsbad, CA, USA) was used to detect ROS levels. Cardiomyocytes were incubated with a working solution (diluting the MitoSOX Red Superoxide Indicator with cell culture medium to 1:2000) in a humidified culture incubator under 5% CO_2_ at 37°C for 10 min. Images with red fluorescence (λex = 510/λem = 580 nm) were captured by a fluorescence microscope (Olympus, Japan).

#### Enzyme-linked immunosorbent assay (ELISA)

Mouse serum samples were collected and stored at −80°C. The concentration of CK-MB (MAK116, Sigma, St. Louis) and cTnT (MAB18742-100, Novus Biologicals) in the serum of mice was measured via enzyme-linked immunosorbent assay kits in accordance with the manufacturer’s instructions. Briefly, samples were seeded in 96-well plates provided by the ELISA kits. Following testing was performed as per the protocols contained in the ELISA kit.

#### Electron microscopy

The ultrastructure of cardiomyocytes was observed using a transmission electron microscope. Briefly, the hearts of mice from each experimental group were perfused and fixed with tube-buffered formaldehyde-glutaraldehyde. The left ventricular myocardium was extracted from the middle of the ventricle and cut into 1 mm^3^ piece. The blocks were fixed in a 10:1 liquid/tissue ratio and incubated overnight at 4 °C. To further process the myocardial mass, the tissues were incubated in 2% sucrose (pH 7.4), 1% OsO4, and 1.5% K_3_[Fe(CN)_6_]·3H_2_O buffer overnight at 22–24 °C. After this, the tissues were dehydrated using graded ethanol and propylene oxide, and finally encapsulated in Epon/Araldite. An RMC-MTXL ultramicrotome and a diatom diamond knife were used to obtain sections. Images were acquired using a CM-120 transmission electron microscope (Philips, The Netherlands). At least 10 fields were observed in each mouse heart sample.

#### Mitochondrial isolation

Mitochondria were isolated from mice heart by using a tissue mitochondrial isolation kit (Beyotime Biotechnology, China, #C3606) according to the manufacturer’s instructions.

#### Mitochondrial respiratory capacity

Mitochondrial respiratory capacity of cardiomyocytes was measured by the oxygen consumption rates (OCRs). Briefly, the DIC mouse adult primary cardiomyocytes were seeded in the Seahorse plate. Cells were analyzed under the XFe96 extracellular flux analyzer (Seahorse Bioscience, Billerica, MA, USA) by adding oligomycin A (1 μM), 1 μM FCCP, antimycin A (1 μM), and rotenone (1 μM).

#### Mitochondrial membrane potential analysis

The mitochondrial membrane potential of myocardial tissue and cardiomyocytes was analyzed by an enhanced mitochondrial membrane potential assay kit with JC-1 (Beyotime Biotechnology, China) according to the manufacturer’s protocol. The myocardial tissue mitochondria were extracted from differently treated mice and analyzed by flow cytometry to detect the mitochondrial membrane potential of myocardial tissue. Flow cytometric analysis was performed on the LSRFORTESSA and FACSAria instruments (BD Biosciences, San Jose, CA, USA) and analyzed using FlowJo software (Tree Star). The mitochondrial membrane potential of cardiomyocytes with red fluorescence was captured by a fluorescence microscope (Olympus, Japan).

#### ATP detection

The ATP production of myocardial tissue was analyzed by an enhanced ATP assay kit (Beyotime Biotechnology, China) according to the manufacturer’s protocol. The results were measured by a microplate reader (BioTek).

#### RNA extraction and real-time qPCR

Total RNA was extracted from tissues and cells using the TRIzol reagent (#15,596,026, Invitrogen) and 2 μg of this RNA were then reverse-transcribed into cDNA using the Prime-Script RT kit (#RR036A, TaKaRa). PCR amplification was performed using the CFX96 real-time PCR (PCR) system (Bio-Rad Laboratories, Inc., CA, USA). The total reaction volume was 10 μL and included 5 μL SYBR Green, 1 μL cDNA, 0.5 μL forward primer, 0.5 μL reverse primer, and 3 μL ddH_2_O. The following two-step PCR amplification protocol was used: 39 cycles of 95 °C for 30 s, 95 °C for 5 s, and 60 °C for 30 s. Relative gene expression was normalized to that of β-actin or 18s RNA using the standard 2^−ΔΔCt^ quantification method. Primer sequences are detailed in Tables S1 and S2.

#### m6A dot blot

Total RNAs from all experimental groups were quantitatively diluted to the same concentration and heated at 95 °C for 3 min. Next, 2 μL of diluted total RNA were evenly distributed onto a Hybond-N+ membrane (#RPN203B, GE Healthcare) and cross-linked with a Stratalinker 2400 UV Crosslinker (1,200 μJ, 5 min). The membrane was blocked with 5% BSA and incubated overnight at 4 °C with anti-m6A antibody (#56593, CST). The membrane was then incubated for 2 h with a secondary antibody, developed, and imaged.

#### MeRIP-seq and MeRIP-seq-qPCR

MeRIP-seq and MeRIP-seq-qPCR services were provided by OE Biotech Inc. (Shanghai, China). Total RNAs from ALKBH5^flox/flox^ (n = 3, ALKBH5^flox/flox^-DOX group) and ALKBH5^flox/flox, αMyHC−Cre^ (n = 3, ALKBH5^flox/flox, αMyHC−Cre^-DOX group) myocardial mouse tissues were extracted using the TRIzol reagent. Each m6A-seq biological replicate used 400 μg of total RNA and yielded approximately 10 μg of double poly(A)-selected RNA. The fragmented RNAs were bound to m6A-Dynabeads. RNA (100 ng of input and 100 ng of post m^6^A-IP positive fraction) was used for library construction using Illumina TruSeq Stranded mRNA protocol. Raw data (raw reads) in fastq format were processed using the Trimmomatic software. Clean data (clean reads) were obtained by removing adapters, poly N, and low-quality reads from the raw data. Next, 250,000 paired reads were randomly extracted from the clean data and aligned against the National Center for Biotechnology Information’s nucleotide database using the BLAST software (ftp://ftp.ncbi.nih.gov/blast/db). The best results (e value < 1e-10 and coverage >80%) were selected. The Sort-MeRNA software was used to remove reads mapping to rRNA and the remaining clean reads were mapped to the reference genome using HISAT2 with default parameters. Unique reads with high mapping quality were retained. The m^6^A-enriched peaks in each m^6^A immunoprecipitation sample were identified using MeTDiff peak calling software, with the corresponding input sample serving as a control. The differential m^6^A-Seq analysis identified differences in RNA methylome in a case-control study. GO and KEGG pathway enrichment analyses of peaks were performed in R based on the hypergeometric distribution. MEME and DREME were used to detect sequence motifs and the Tomtom software was used to annotate the motifs. MeRIP-qPCR and subsequent RNA immunoprecipitation qPCR (RIP-qPCR) were performed using the RIP kit (Millipore, MA, USA) and m^6^A (MeRIP-qPCR) or ALKBH5 (RIP-qPCR) antibodies, according to the manufacturer’s instructions. Briefly, myocardial tissue was lysed with RIP buffer and immunoprecipitated with specific or control IgG antibodies overnight at 4 °C, followed by RNA purification. The immunoprecipitated RNA was analyzed using RT-qPCR. Primer sequences are shown in Table S2.

#### Measurement of Rasal3 mRNA stability

ALKBH5-KI adult and control mouse cardiomyocytes were extracted using the method described above and treated with actinomycin D (5 μg/mL, Sigma-Aldrich) for 0, 2, 4, or 6 h. RNA was then extracted and diluted to give a final concentration of 100 ng/μL. Rasal3 mRNA levels were quantified by RT-qPCR. Rasal3 mRNA levels at 0 h were used as controls. Relative mRNA decay rates and half-lives were determined using non-linear regression (single-phase decay) in GraphPad Prism 8.0 (San Diego, CA, USA).

#### Dual-luciferase reporter assay

Wild-type Rasal3 5′-UTR, coding region (CDS), 3′-UTR, and Rasal3 mutant (MUT, 3′-UTR mutant) sequences were amplified by PCR and inserted into the pGL3 vectors (Beyotime): Rasal3-5-UTR, Rasal3-CDS, Rasal3-3′-UTR, and Rasal3-MUT, respectively. 293T cells were seeded into 24-well plates and cultured overnight. The Lipofectamine 3000 Reagent (Invitrogen) was used to co-transfect 293T cells with empty vector, Rasal3-5-UTR, Rasal3-CDS, Rasal3-3′-UTR, and Rasal3-MUT with Ad-OE-Control or Ad-OE-ALKBH5, and Renilla luciferase plasmids. Cells were harvested and lysed after 48 h. Firefly luciferase activity was detected using the Dual-Luciferase Reporter Assay System (Promega) and normalized to the Renilla luciferase control levels.

#### dCas13b-ALKBH5 and guide RNAs (gRNAs)

The dCas13b-ALKBH5 plasmid, gRNA plasmid and non-targeting gRNA (NT-gRNA) plasmid were kindly supplied by professor Hongsheng Wang.^60^ The gRNAs (gRNA 1–3) targeting the sequence about 100–200 nt away from m^6^A sites of Rasal3 mRNA were designed and listed in in Table S3. The plasmid transfection was performed with Lipofectamine 3000 (Invitrogen) following the manufacturer’s protocol. For six-well assays, AC16 cells were transfected with 1.5 μg dCas13b-ALKBH5 and 1.5 μg gRNAs and cultured for 48 h.

#### Calcein/PI live/dead viability test

Adult mouse cardiomyocytes were added to Calcein AM/PI detection working solution (Calcein/PI Cell Viability/Cytotoxicity Assay Kit, Beyotime) and incubated in the dark at 37 °C for 30 min. The staining effect was observed under a fluorescence microscope immediately after incubation (Calcein AM generates green fluorescence, Ex/Em = 494–517 nm; PI generates red fluorescence, Ex/Em = 535–617 nm).

#### Western blot assay

Cardiac muscle tissue was harvested and placed in RIPA lysis buffer containing 1 mM phenylmethanesulfonyl fluoride. Protein samples were separated using 10% and 15% SDS-PAGE and transferred to polyvinylidene difluoride membranes (Biotech Well). The membranes were blocked with 5% BSA in TBST for 2 h and incubated overnight at 4 °C with the following primary antibodies: anti-ALKBH5 (ab195377, Abcam), anti-Mettl3 (ab195352, Abcam), anti-Mettl14 (ab252562, Abcam), anti-FTO (ab280081, Abcam), anti-BAX (ab3191, Abcam), anti-BCL-2 (ab196495, Abcam), anti-cleaved caspase3 (ab214430, Abcam), anti-Raf1 (A0223, Abclone), anti-phospho-Raf1-S259 (AP1012, Abclone), anti-p44/42 ERK1/2 (4370S, CST), anti-FLAG (Abcam, ab1162), anti-Rasal3 (NBP2-83439, Novusbio), and anti-β-actin (4970S, CST). The samples were then incubated at room temperature (24 °C) for 1.5 h with horseradish peroxidase-conjugated secondary antibody. Proteins were detected using Immobilon Western Chemiluminescent HRP Substrate (Millipore, Billerica, MA, USA) and gel images were captured using ImageQuant LAS 4000 Mini Biomolecular Imager (GE Healthcare, Barrington, IL, USA).

#### Active Ras pull-down test

Ras activity was detected using the Ras Activation Assay Kit (Bioyears) following the manufacturer’s instructions, using 500 μg of protein per condition. The quantity of RAS-GTP pulled down by Raf1 RBD (RAS-binding domain) relative to total Ras expression as determined using western blotting reflects Ras activity.

### Quantification and statistical analysis

Data are expressed as mean ± SEM. Statistical analyses were conducted using the GraphPad Prism 8.0 software (GraphPad Software, San Diego, CA). The normality of data distribution was tested using the Kolmogorov-Smirnov test. Mann-Whitney U test was used when the group data were not normally distributed or if group variances were unequal. The homogeneity of variance was analyzed using Levene’s test. Continuous data with normal distribution were assessed using either Student’s *t* test, one-way ANOVA with a post hoc test or two-way ANOVA followed by a post hoc test (Tukey-Kramer). The results were considered significant when p < 0.05. The meaning of asterisks number were ∗p < 0.05, ∗∗p < 0.05, ∗∗∗p < 0.001, ∗∗∗∗p < 0.0001.

## Data Availability

All data reported in this paper will be shared by the lead contact upon request. This paper does not report original code. Any additional information required to reanalyze the data reported in this paper is available from the lead contact upon request.
