# Supplementary material for: m6A demethylase ALKBH5 attenuates doxorubicin-induced cardiotoxicity via posttranscriptional stabilization of Rasal3
Source: iScience. 2023 Feb 16;26(3):106215. doi: 10.1016/j.isci.2023.106215 (PMC9982307; doi:10.1016/j.isci.2023.106215)
Supplement: Document S1. Figures S1–S17 and Tables S1–S3 [file mmc1.pdf]

iScience, Volume *26*

Supplemental information

m^6A demethylase ALKBH5 attenuates doxorubicin-induced cardiotoxicity via posttranscriptional stabilization of Rasal3

Ri-Feng Gao, Kun Yang, Ya-Nan Qu, Xiang Wei, Jia-Ran Shi, Chun-Yu Lv, Yong-Chao Zhao, Xiao-Lei Sun, Ying-Jia Xu, and Yi-Qing Yang

Figure S1

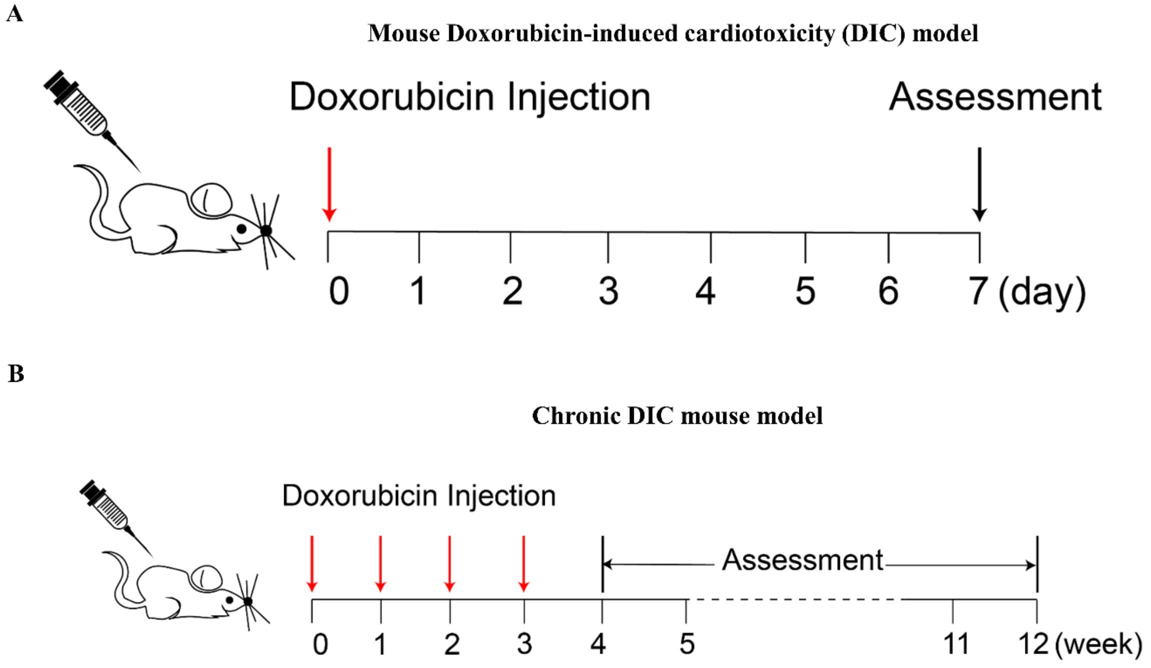

Figure S1. Schematic diagram of construction of DIC and CDIC mouse models. Related to the STAR Methods.

(A) Schematic diagram of experimental procedure of acute doxorubicin-induced cardiotoxicity (DIC) in mice (IP, 20mg/kg). (B) Schematics of the experimental procedure for doxorubicin administration to the chronic DIC model mice (IP, 5 mg/kg once weekly for 4 weeks).

Figure S2

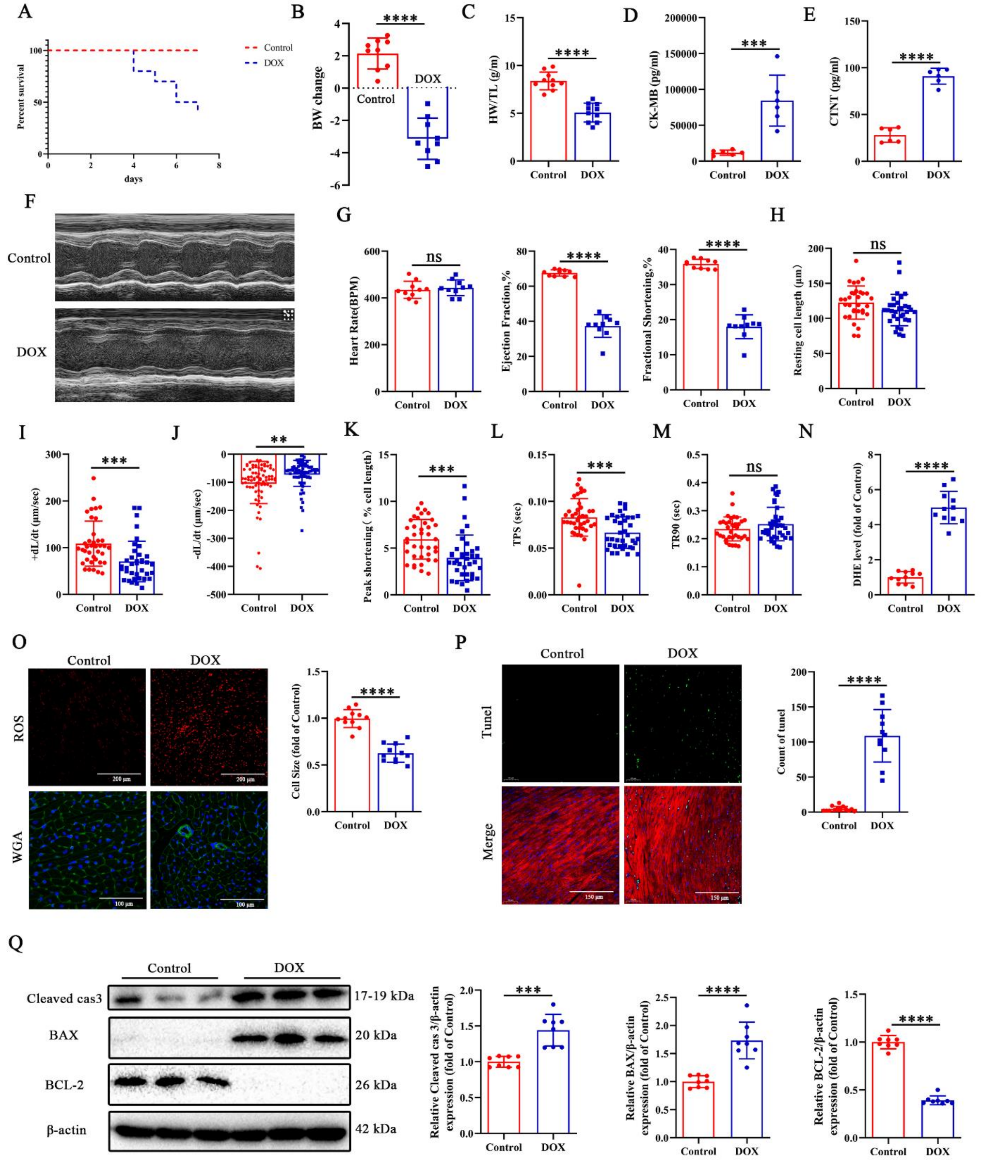

Figure S2. Doxorubicin-induced cardiotoxic injury, down-regulation of ALKBH5 expression in myocardial tissue. Related to the Figure 1.

(A) Kaplan Meier survival curves showing the survival of DOX-stressed (20mg/kg) mice (n=20). (B) Body weight change during the DIC experiment (n=9). (C) Ratio of heart weight to tibial length after DIC (n=10). Detection of cardiotoxicity indexes CK-MB (D) and CTnT (E) by ELISA (n=6). (F) Representative images of echocardiography tracing in DIC mice. (G) Heart rate (BPM), ejection fraction and fractional shortening (n=10). (H) Resting cell length (n＞10). (I) Maximal velocity of re-lengthening (+dL/dt; n＞10). (J) Maximal velocity of shortening (-dL/dt; n＞10). (K) Peak shortening (PS, normalized to resting cell length; n＞10). (L) Time-to-PS (TPS; n＞10). (M) Time-to-90% re-lengthening (TR90; n＞10). (N and O) Relative index of excessive oxygen species (ROS) fluorescence (Bar=200 μm; n＞6). (O) Cardiomyocyte cell size measurement by wheat germ agglutinin (WGA) staining of heart (Bar=200 μm). (P) Apoptosis measured by TUNEL (terminal deoxynucleotidyl transferase dUTP nick end labeling) staining in heart sections (Bar=150 μm; n＞6). (Q) Representative western blot of Cleaved caspase-3, BAX and BCL-2 levels in DIC (n＞6). Data are depicted as the mean ± SEM. Statistical significance was determined by student's t test or one-way ANOVA with a post-hoc Holm-Sidak test, ns, not significant; *P<0.05; **P<0.05; ***P<0.001; ****P<0.0001.

Figure S3

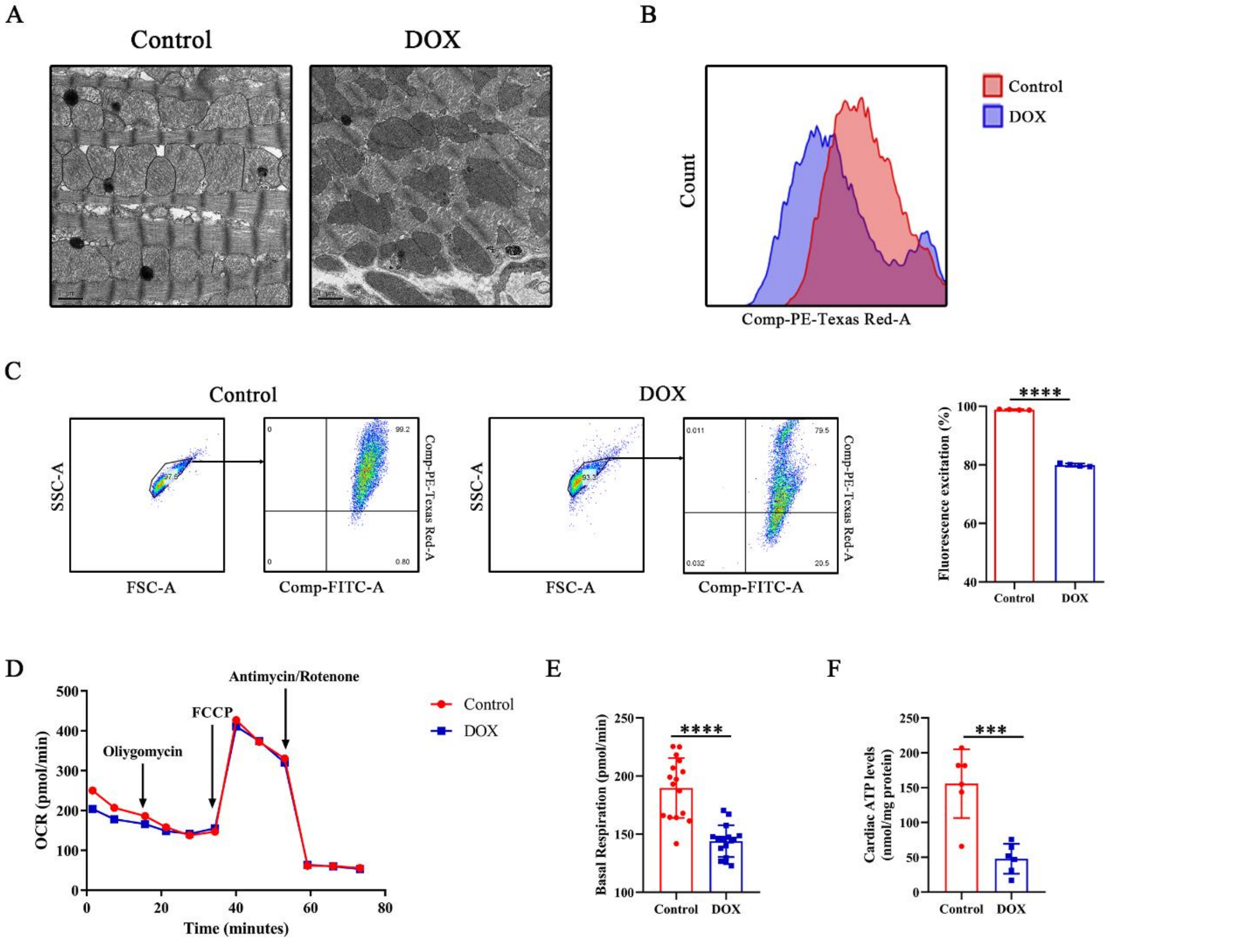

Figure S3. Doxorubicin-induced cardiac mitochondrial dysfunction. Related to the Figure 1. (A) Representative images of mitochondrial morphology in myocardial tissue with or without DOX (Bar=1 μm). (B and C) JC-1 changes in DIC mice. (D) OCRs changes of cardiomyocytes in DIC mice. (E) Statistical analysis of basal respiration in DIC mice. (F) ATP production of myocardial tissue in DIC mice. Data are depicted as the mean ± SEM. Statistical significance was determined by student's t test, ns, not significant; *P<0.05; **P<0.05; ***P<0.001; ****P<0.0001.

Figure S4

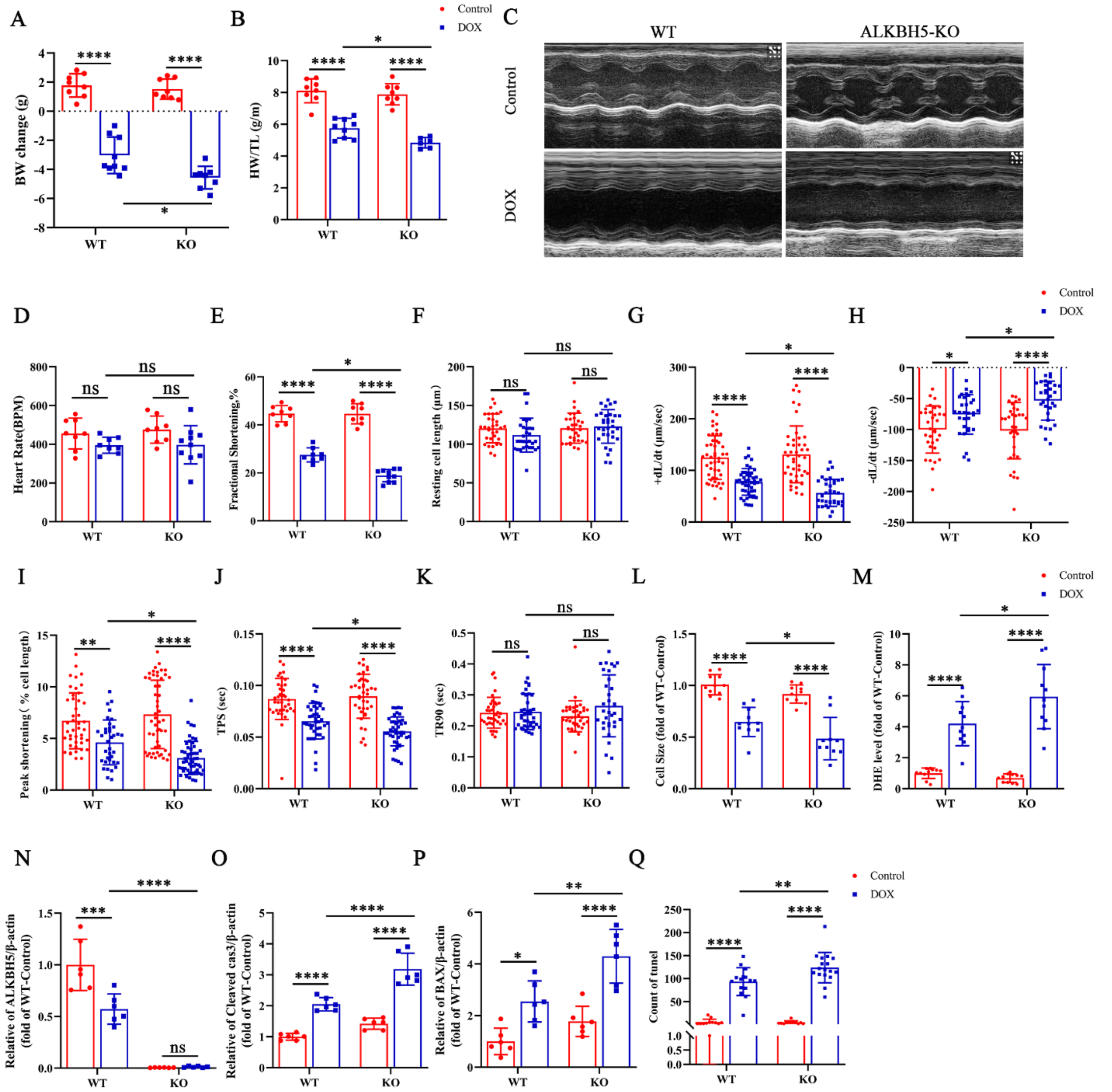

Figure S4. ALKBH5-KO aggravates DIC injury. Related to the Figure 1.

(A) Body weight change during the DIC experiment in ALKBH5-KO and control mice (n＞6). (B) Ratio of heart weight to tibial length after DIC (n=6-10). (C) Representative images of echocardiography tracing in ALKBH5-KO and WT mice. (D) Heart rate (BPM) and (E) fractional shortening (n＞6). (F) Resting cell length (n＞10). (G) Maximal velocity of re-lengthening (+dL/dt;

n＞10). (H) Maximal velocity of shortening (-dL/dt; n＞10). (I) Peak shortening (PS, normalized to resting cell length; n＞10). (J) Time-to-PS (TPS; n＞10). (K) Time-to-90% re-lengthening (TR90; n＞10). The analysis of WGA (L; n＞6) and ROS (M; n＞6) staining of ALKBH5-KO and WT mice in DIC model. Western blot analysis of ALKBH5 (N; n=6), Cleaved caspase-3 (O; n=6) and BAX (P; n=6) in ALKBH5-KO and WT mice in DIC model. (Q) Count of tunel staining in WT and ALKBH5-KO heart sections (n＞6). Data are depicted as the mean ± SEM. Statistical significance was determined by two-way ANOVA with a post-hoc Holm-Sidak test, ns, not significant; *P<0.05; **P<0.01; ***P<0.001; ****P<0.0001.

Figure S5

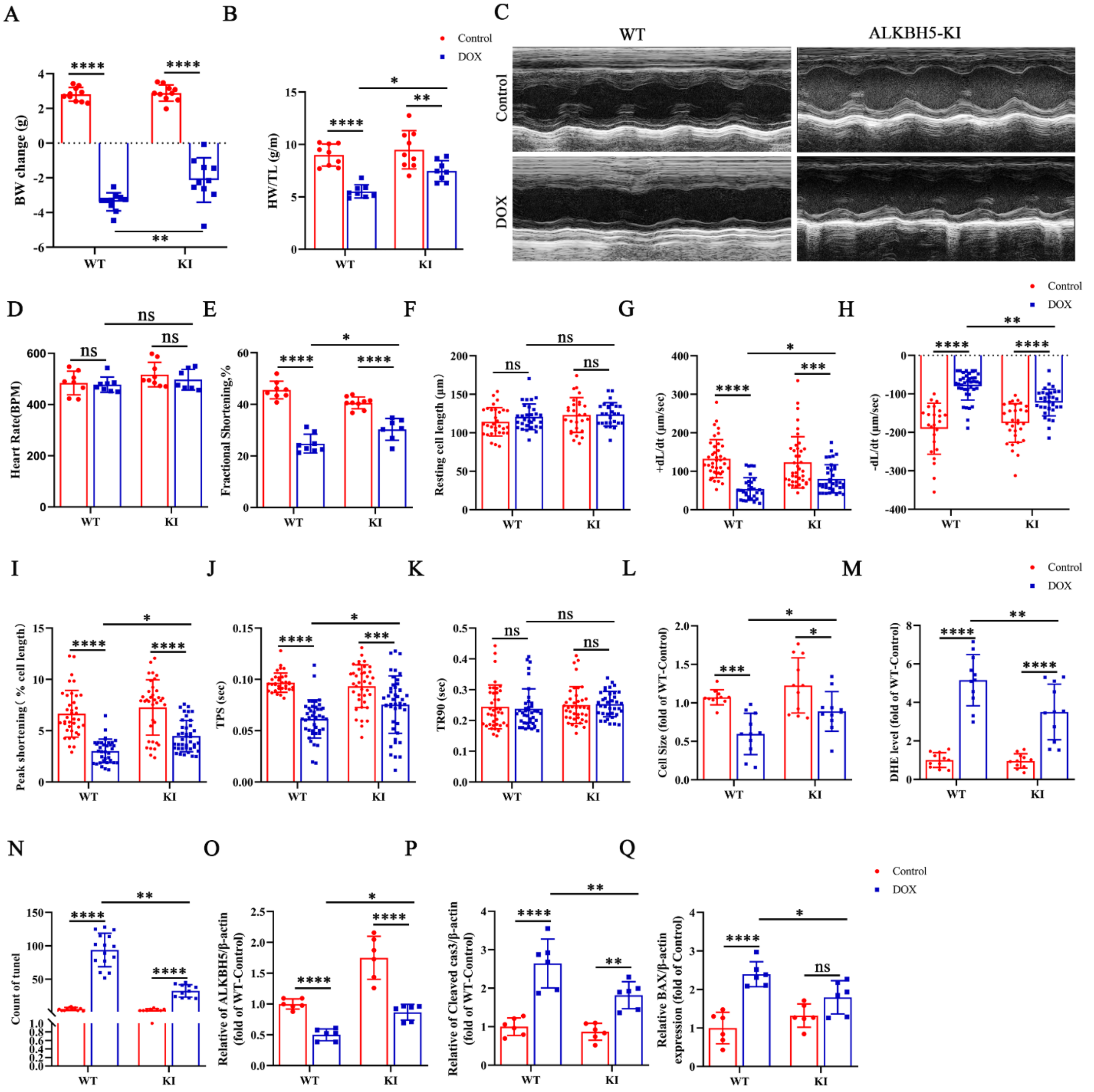

Figure S5. ALKBH5-KI attenuates DIC injury. Related to the Figure 1.

(A) Body weight change during the DIC experiment in ALKBH5-KI and control mice (n＞6). (B) Ratio of heart weight to tibial length after DIC (n=6-10). (C) Representative images of echocardiography tracing in ALKBH5-KI and WT mice. (D) Heart rate (BPM) and (E) fractional shortening (n＞6). (F) Resting cell length (n＞10). (G) Maximal velocity of re-lengthening (+dL/dt; n＞10). (H) Maximal velocity of shortening (-dL/dt; n＞10). (I) Peak shortening (PS, normalized

to resting cell length; n＞10). (J) Time-to-PS (TPS; n＞10). (K) Time-to-90% re-lengthening (TR90; n＞10). The analysis of WGA (L; n＞6) and ROS (M; n＞6) staining of ALKBH5-KI and WT mice in DIC model. Western blot analysis of ALKBH5 (N; n=6), Cleaved caspase-3 (O; n=6) and BAX (P; n=6) in ALKBH5-KO and WT mice in DIC model. (Q) Count of tunel staining in WT and ALKBH5-KI heart sections (n＞6). Data are depicted as the mean ± SEM. Statistical significance was determined by two-way ANOVA with a post-hoc Holm-Sidak test, ns, not significant; *P<0.05; **P<0.01; ***P<0.001; ****P<0.0001.

Figure S6

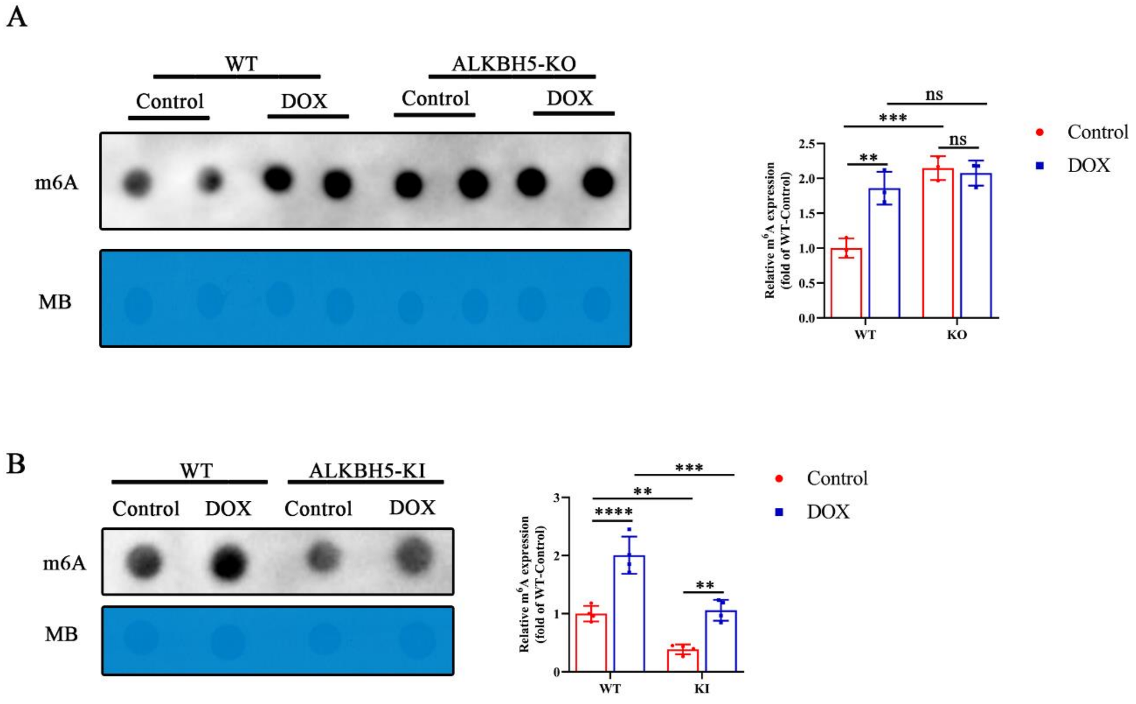

Figure S6. m6A levels in the myocardium of ALKBH5-KO and ALKBH5-KI mice. Related to the Figure 1.

(A) Dot blot showed m6A expression levels in ALKBH5-KO and WT groups (n=3). (B) Dot blot showed m6A expression levels in ALKBH5-KI and WT groups (n=4). Data are depicted as the mean ± SEM. Statistical significance was determined by two-way ANOVA with a post-hoc Holm-Sidak test, ns, not significant; *P<0.05; **P<0.01; ***P<0.001; ****P<0.0001.

Figure S7

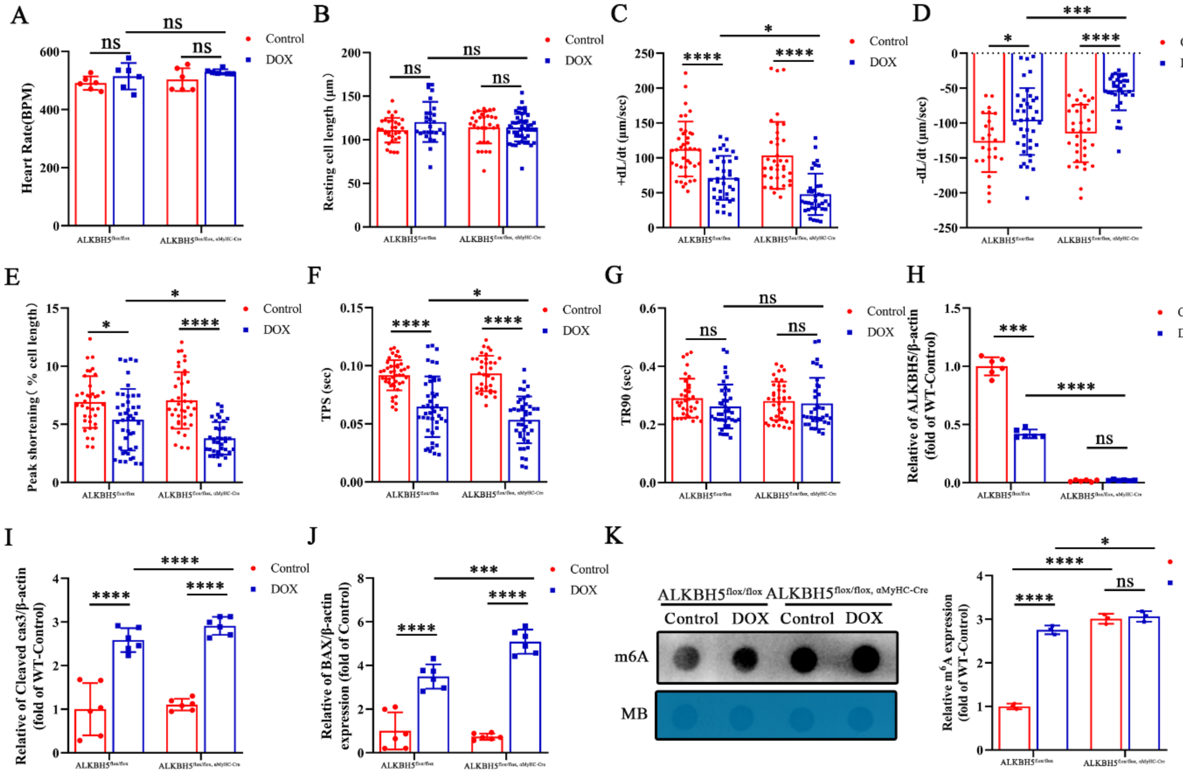

Figure S7. ALKBH5-myocardial specific knockout aggravates DIC injury. Related to the Figure 2.

(A) Heart rate (BPM) of ALKBH5$^{flox/flox}$ and ALKBH5$^{flox/flox,\ \alpha\text{-}MyHC\text{-}Cre}$ mice (n=6).

(B) Resting cell length (n＞10). (C) Maximal velocity of re-lengthening (+dL/dt; n＞10). (D) Maximal velocity of shortening (-dL/dt; n＞10). (E) Peak shortening (PS, normalized to resting cell length; n＞10). (F) Time-to-PS (TPS; n＞10). (G) Time-to-90% re-lengthening (TR90; n＞10). Western blot ALKBH5 (H), Cleaved caspase-3 (I) and BAX (J) analysis of representative ALKBH5$^{flox/flox}$ and ALKBH5$^{flox/flox,\ \alpha\text{-}MyHC\text{-}Cre}$ mice in DIC model (n=6). (K) Dot blot showed m6A expression levels in ALKBH5$^{flox/flox,\ \alpha\text{-}MyHC\text{-}Cre}$ and Control groups (n=3). Data are depicted as the mean ± SEM. Statistical significance was determined by two-way ANOVA with a post-hoc Holm-Sidak test, ns, not significant; *P<0.05; **P<0.01; ***P<0.001; ****P<0.0001; compared with the control group.

Figure S8

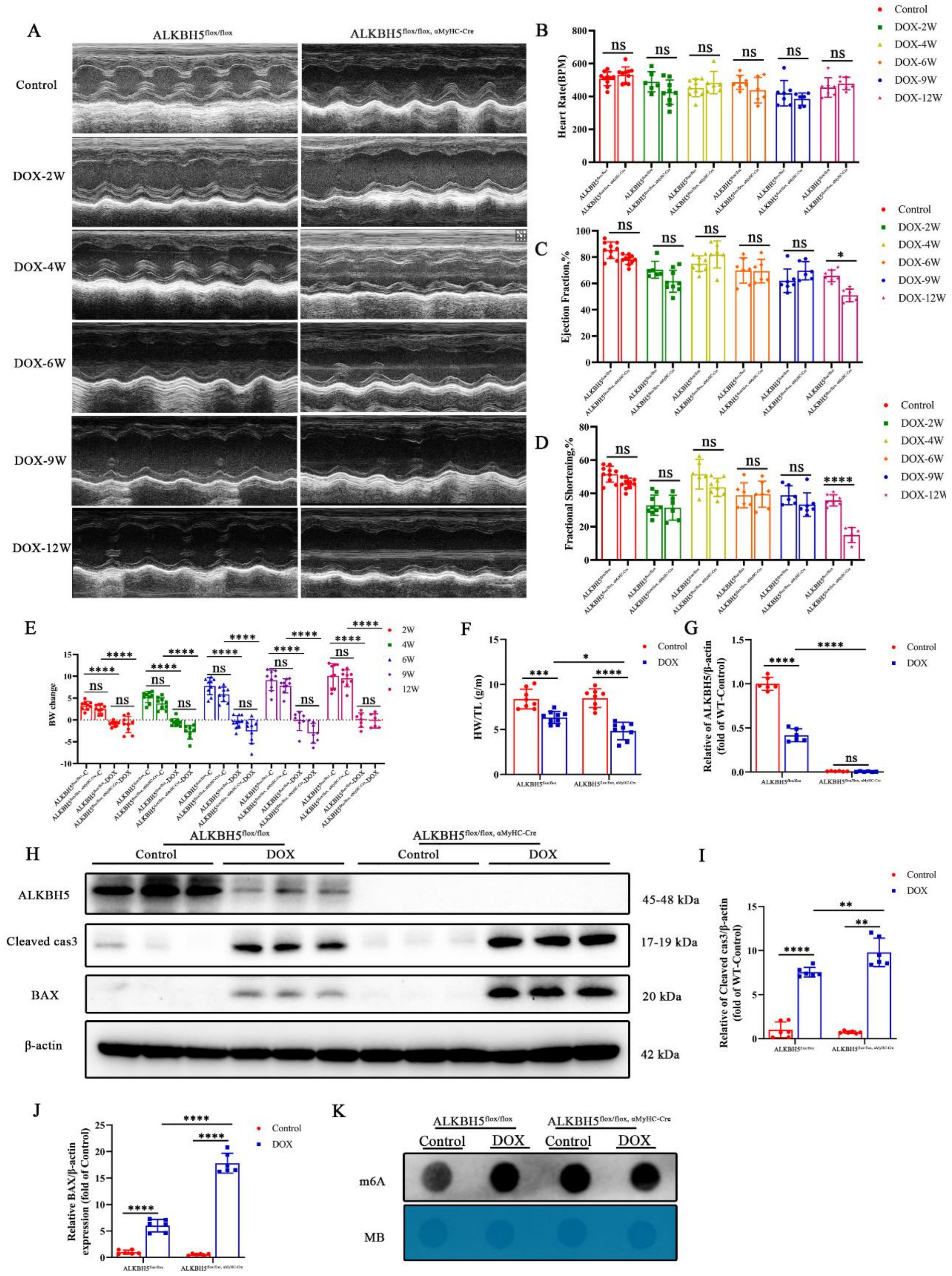

Figure S8. ALKBH5-myocardial specific knockout aggravates CDIC injury. Related to the Figure 2.

(A) Representative images of echocardiography tracing in ALKBH5$^{flox/flox}$ and ALKBH5$^{flox/flox,\ \alpha\text{-}MyHC\text{-}Cre}$ mice. (B) Heart rate (BPM), (C) fractional shortening and (D) fractional shortening of ALKBH5$^{flox/flox}$ and ALKBH5$^{flox/flox,\ \alpha\text{-}MyHC\text{-}Cre}$ mice in CDIC model (n＞5). (E) Body weight change during the CDIC experiment in ALKBH5$^{flox/flox,\ \alpha\text{-}MyHC\text{-}Cre}$ and control mice (n＞6). (F) Ratio of heart weight to tibial length after CDIC (n＞6). (H) Western blot analysis of the apoptosis protein expression levels of ALKBH5 (G), Cleaved caspase-3 (I) and BAX (J) levels in ALKBH5$^{flox/flox,\ \alpha\text{-}MyHC\text{-}Cre}$-DOX and ALKBH5$^{flox/flox}$-DOX (n=6). (K) Representative dot blot images showing m6A abundance after injection under normal saline and DOX. Data are depicted as the mean ± SEM. Statistical significance was determined by two-way ANOVA with a post-hoc Holm-Sidak test, ns, not significant; *P<0.05; **P<0.01; ***P<0.001; ****P<0.0001.

Figure S9

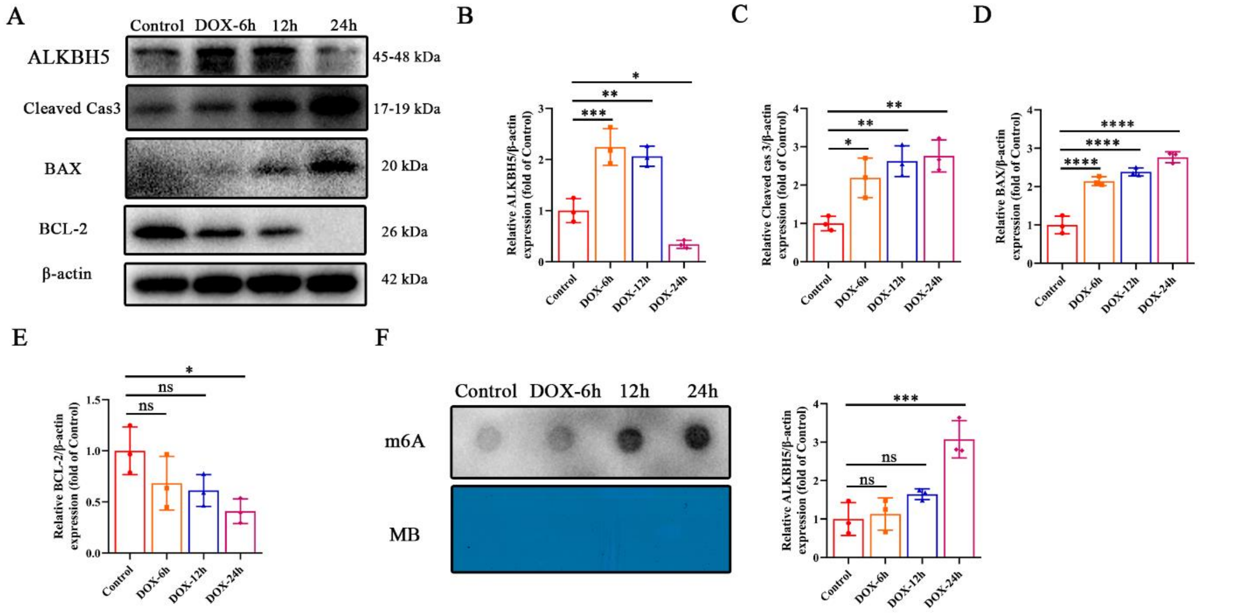

Figure S9. Doxorubicin induces cardiomyocyte apoptosis, and the expression of ALKBH5 in cardiomyocytes fluctuates. Related to the Figure 3.

(A) Western blot analysis of the apoptosis proteins expression levels of ALKBH5 (B), Cleaved caspase-3 (C), BAX (D) and BCL-2 (E) levels in adult mouse cardiomyocytes at different time points after DOX intervention (n=3). (E) Dot blot reveals m6A levels in adult mouse cardiomyocytes at different time points after DOX intervention (n=3). Data are depicted as the mean ± SEM. Statistical significance was determined by student's t test or one-way ANOVA with a post-hoc Holm-Sidak test, ns, not significant; *P<0.05; **P<0.05; ***P<0.001; ****P<0.0001; compared with the control group.

Figure S10

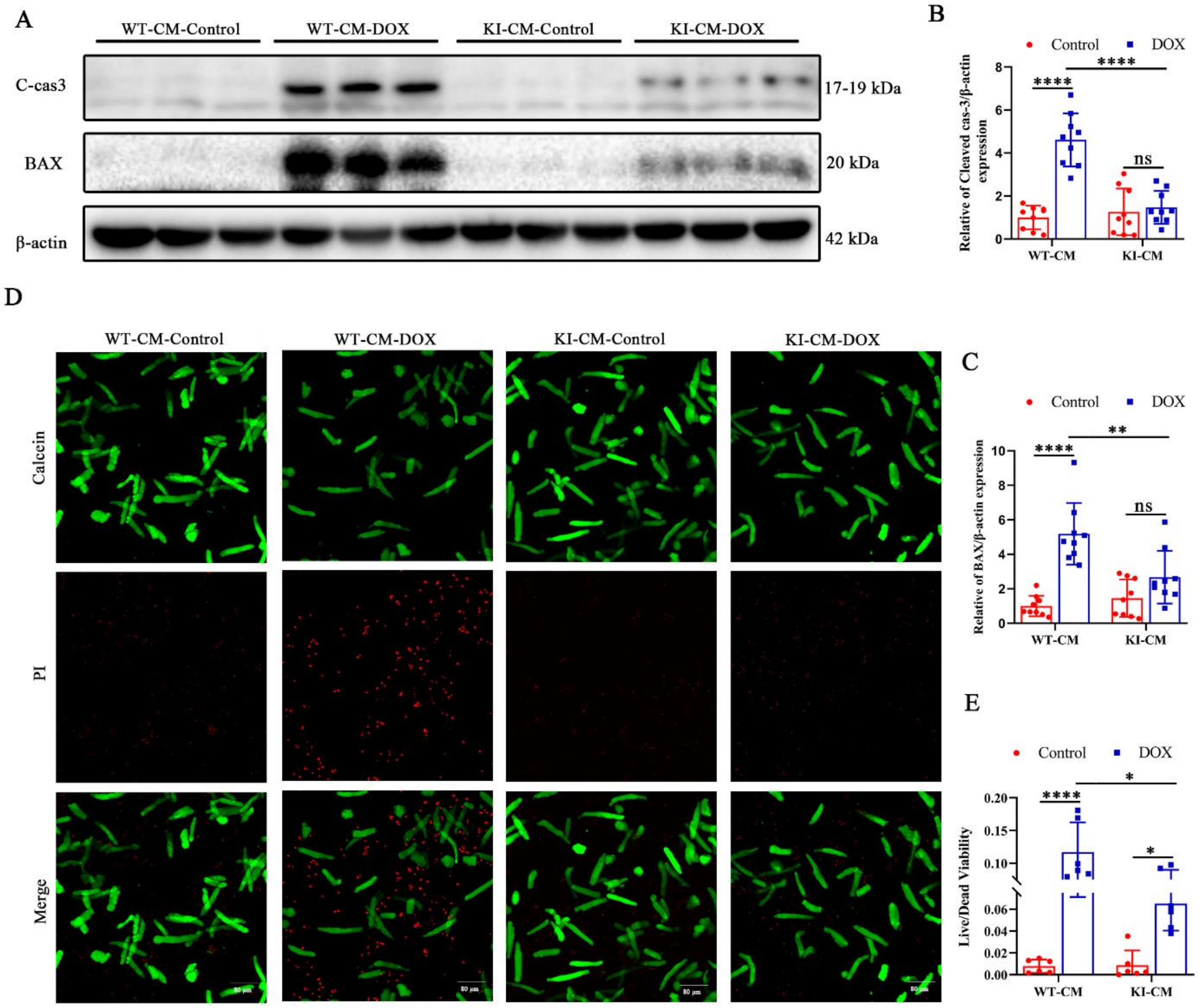

Figure S10. ALKBH5 overexpression alleviates DOX-induced cardiomyocyte dysfunction. Related to the Figure 3.

Western blot analysis of the apoptosis protein expression levels of ALKBH5, Cleaved caspase-3 (A, B) and BAX (A, C) levels in ALKBH5-KI-CM and WT-CM after DOX treatment (n＞6). (D and E) Calcein/PI-Live/Dead staining in ALKBH5-KO-CM and WT-CM after DOX treatment (Bar=80 μm; n=6). Data are depicted as the mean ± SEM. Statistical significance was determined by student's t test, one-way or two-way ANOVA with a post-hoc Holm-Sidak test, ns, not significant; *P<0.05; **P<0.01; ***P<0.001; ****P<0.0001; compared with the control group.

Figure S11

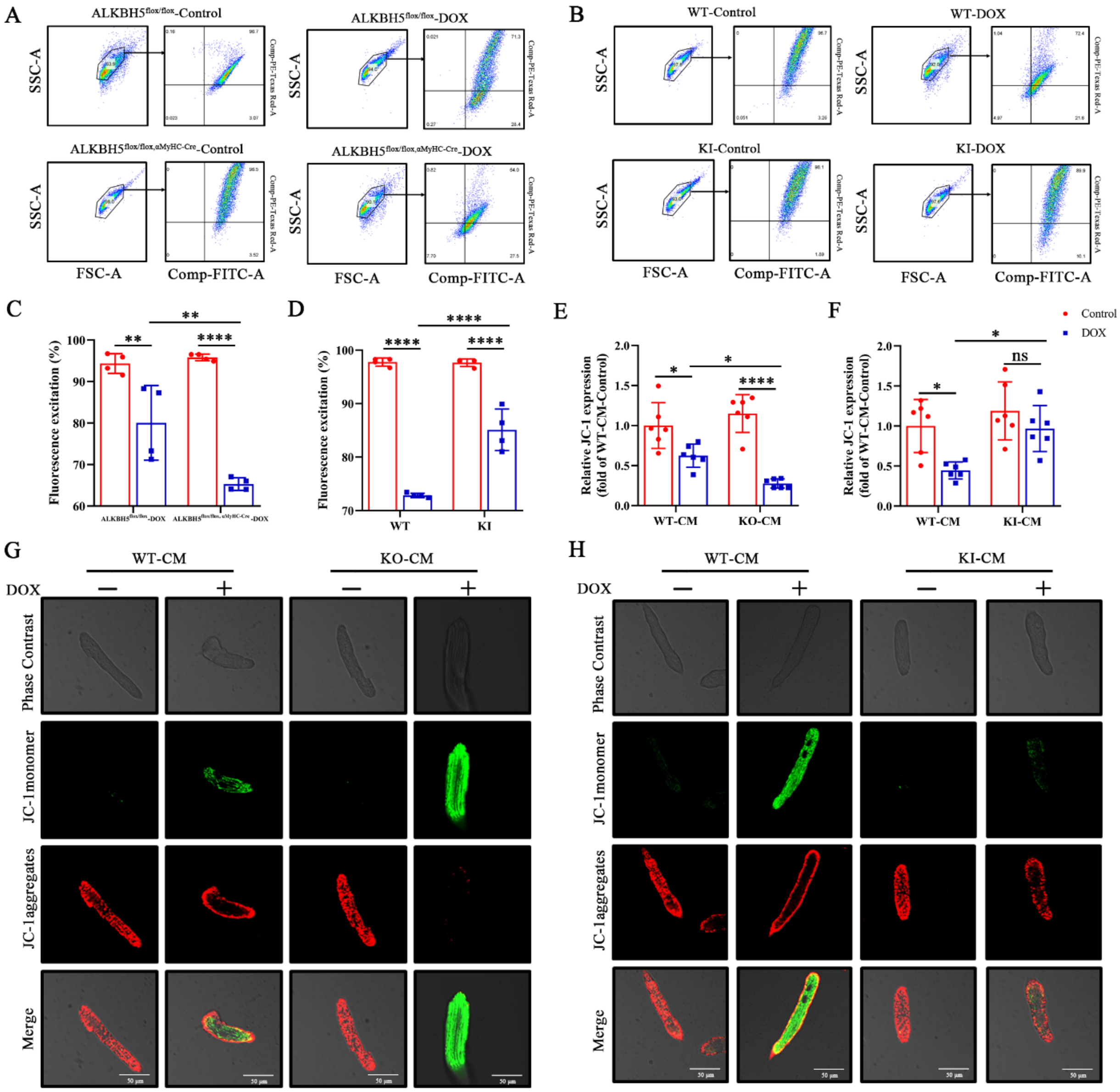

Figure S11. ALKBH5 reduces DOX-induced mitochondrial JC-1 levels. Related to the Figure 4.

(A and C) Flow cytometric detection of mitochondrial membrane potential JC-1 in myocardial tissue of with or without DOX in ALKBH5$^{flox/flox}$ and ALKBH5$^{flox/flox,\ \alpha\text{-}MyHC\text{-}Cre}$ mice (n=4). (B and D) Flow cytometric detection of mitochondrial membrane potential JC-1 in myocardial tissue of with or without DOX in WT and KI mice (n=4). (E and G) JC-1 changes resulting from DOX

treatment in ALKBH5-KO-CM and WT-CM (Bar=50 μm, n=6). (F and H) JC-1 changes resulting from DOX treatment in ALKBH5-KI-CM and WT-CM (Bar=50 μm, n=6). Data are depicted as the mean ± SEM. Statistical significance was determined by student's t test or one-way ANOVA or two-way ANOVA with a post-hoc Holm-Sidak test, ns, not significant; *P<0.05; **P<0.05; ***P<0.001; ****P<0.0001.

Figure S12

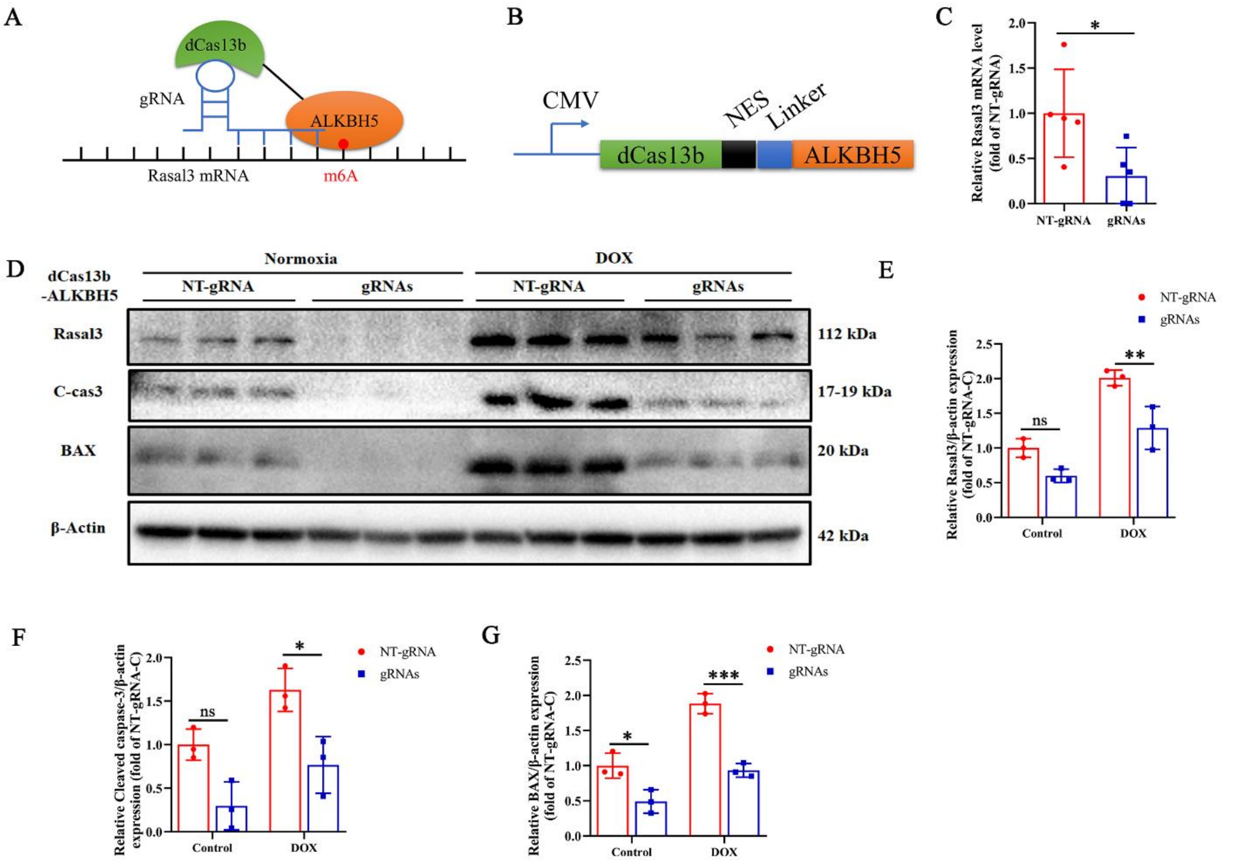

Figure S12. ALKBH5 exerts cardio-protection by mediating m6A demethylation of Rasal3 Mrna. Related to the Figure 5.

The construction strategy (A) and working diagram (B) of dCas13b-ALKBH5. (C) The mRNA levels of Rasal3 were measured by RT-qPCR. (D) The protein levels of Rasal3 (E), Cleaved caspase-3 (F) and BAX (G) were measured by Western blot (n=3). Data are depicted as the mean ± SEM. Statistical significance was determined by student's t test or one-way ANOVA or two-way ANOVA with a post-hoc Holm-Sidak test, ns, not significant; *P<0.05; **P<0.05; ***P<0.001; ****P<0.0001.

Figure S13

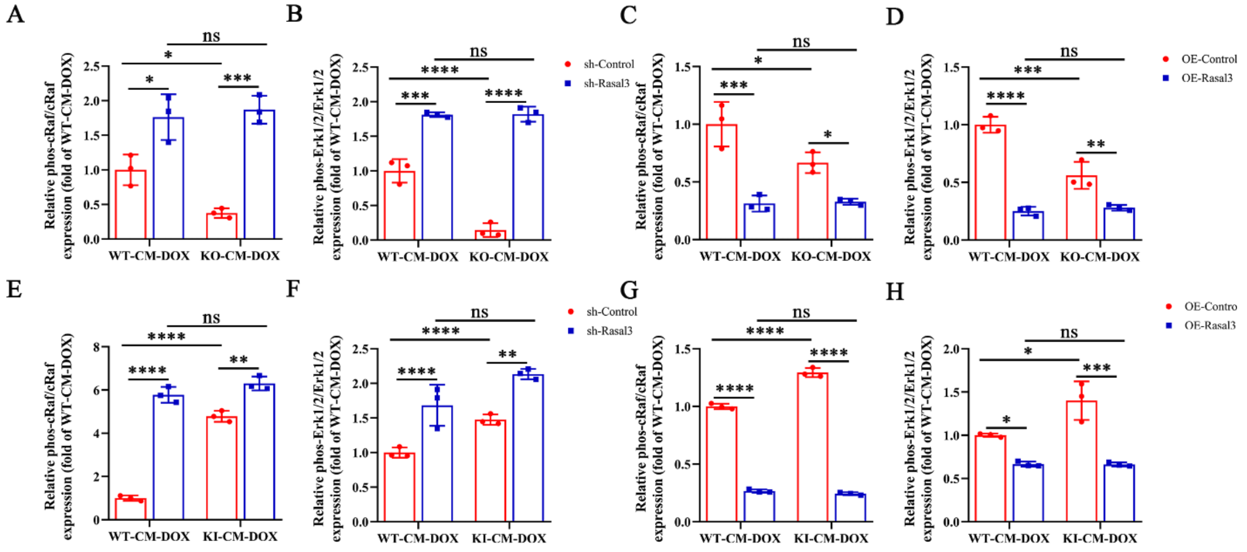

Figure S13. ALKBH5 exerts cardioprotective effects by promoting RAS/RAF/ERK signaling via m6A demethylation of Rasal3 mRNA. Related to the Figure 6.

Western blot analysis of phosphorylated cRaf (phos-cRaf; A), and phosphorylated Erk (phos-Erk1/2; B) in ALKBH5-KO-CM-DOX and WT-CM-DOX after shRNA-Rasal3 knockdown (n=3). Western blot analysis of phosphorylated cRaf (phos-cRaf; C), and phosphorylated Erk (phos-Erk1/2; D) in ALKBH5-KO-CM-DOX and WT-CM-DOX after OE-Rasal3 overexpression (n=3). Western blot analysis of phosphorylated cRaf (phos-cRaf; E), and phosphorylated Erk (phos-Erk1/2; F) in ALKBH5-KI-CM-DOX and WT-CM-DOX after shRNA-Rasal3 knockdown (n=3). Western blot analysis of phosphorylated cRaf (phos-cRaf; G), and phosphorylated Erk (phos-Erk1/2; H) in ALKBH5-KI-CM-DOX and WT-CM-DOX after OE-Rasal3 overexpression (n=3). Data are depicted as the mean ± SEM. Statistical significance was determined by student's t test or one-way ANOVA or two-way ANOVA with a post-hoc Holm-Sidak test, ns, not significant; *P<0.05; **P<0.05; ***P<0.001; ****P<0.0001.

Figure S14

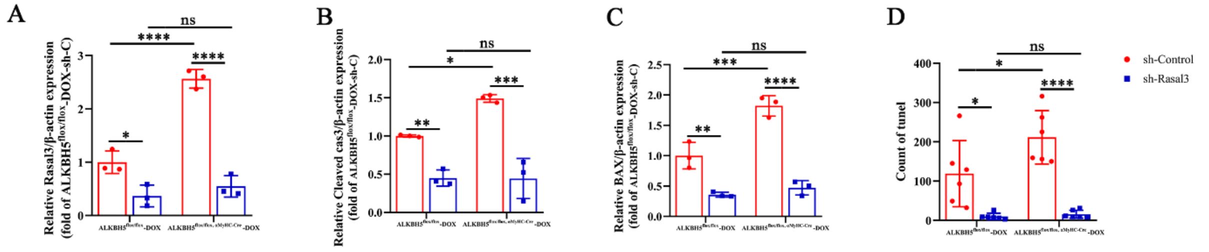

Figure S14. Adenovirus knockdown of Rasal3 can antagonize the effect of ALKBH5 and alleviate DIC injury. Related to the Figure 7.

Western blot analysis of Rasal3 (A), Cleaved caspase-3 (B) and BAX (C) in ALKBH5$^{flox/flox}$ and ALKBH5$^{flox/flox,\ \alpha\text{-}MyHC\text{-}Cre}$ after pAAV-shRNA-Rasal3 knockdown (n=3). (D) Count of TUNEL in ALKBH5$^{flox/flox}$ and ALKBH5$^{flox/flox,\ \alpha\text{-}MyHC\text{-}Cre}$ after pAAV-shRNA-Rasal3 knockdown (n=3）. Data are depicted as the mean ± SEM. Statistical significance was determined by student’s t test or one-way ANOVA or two-way ANOVA with a post-hoc Holm-Sidak test, ns, not significant; *P<0.05; **P<0.05; ***P<0.001; ****P<0.0001.

Figure S15

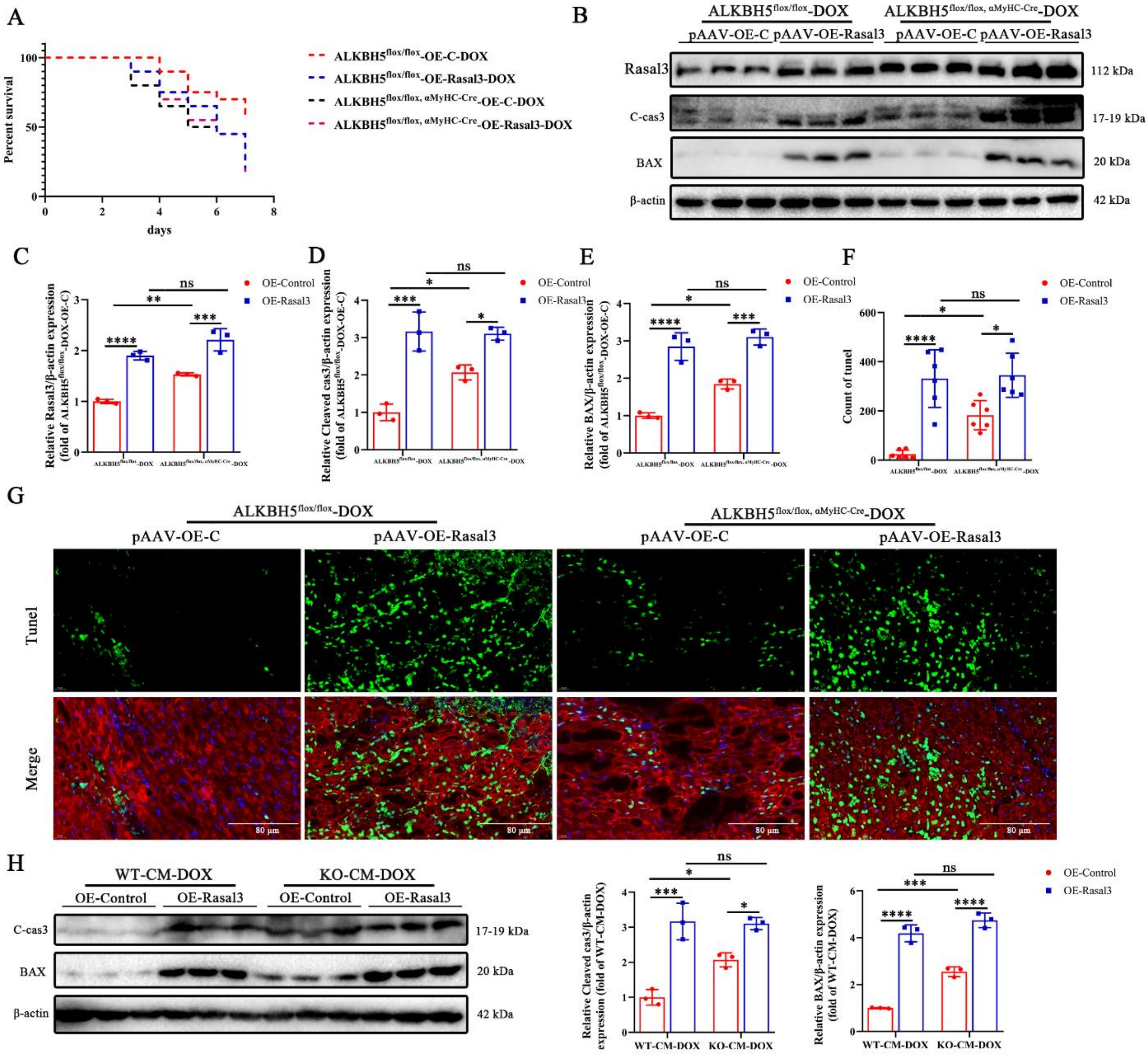

Figure S15. Adenovirus knockdown of Rasal3 alleviate DIC injury in ALKBH5-KI Mice. Related to the Figure 7.

(A) Kaplan Meier survival curves showing the survival of DOX-stressed mice in WT and ALKBH5-KI after pAAV-shRNA-Rasal3 knockdown (n=20）. (B) Representative Western blots of Rasal3, Cleaved caspase-3 and BAX in WT and ALKBH5-KI after pAAV-shRNA-Rasal3 knockdown (n=3）. Western blot analysis of Rasal3 (C), Cleaved caspase-3 (D) and BAX (E) in WT and KI mice after pAAV-shRNA-Rasal3 knockdown (n=3）. (F) Count of TUNEL in WT

and KI mice after pAAV-shRNA-Rasal3 knockdown (n=3）. (G) Apoptosis measured by TUNEL staining in WT and ALKBH5-KI heart sections (Bar=80 μm). (H) Western blot analysis of Cleaved caspase-3 and BAX in ALKBH5-KI-CM-DOX and WT-CM-DOX after pAAV-shRNA-Rasal3 overexpression. Data are depicted as the mean ± SEM. Statistical significance was determined by student's t test or one-way ANOVA or two-way ANOVA with a post-hoc Holm-Sidak test, ns, not significant; *P<0.05; **P<0.05; ***P<0.001; ****P<0.0001.

Figure S16

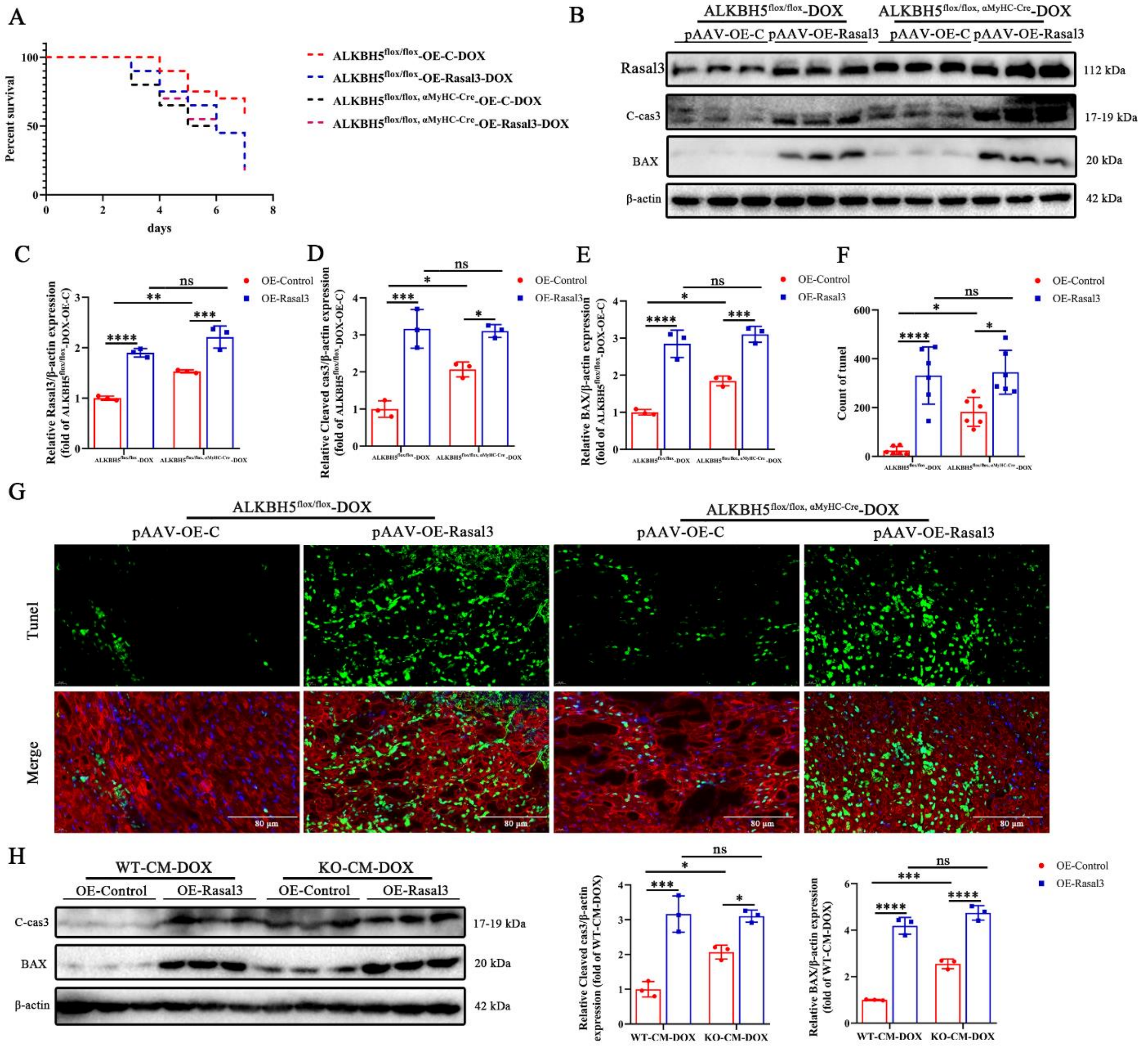

Figure S16. Rasal3 adenovirus overexpression aggravates DIC injury in ALKBH5$^{flox/flox,\ \alpha\text{-}MyHC\text{-}Cre}$ Mice. Related to the Figure 7.

(A) Kaplan Meier survival curves showing the survival of DOX-stressed mice in ALKBH5$^{flox/flox}$ and ALKBH5$^{flox/flox,\ \alpha\text{-}MyHC\text{-}Cre}$ after pAAV-OE-Rasal3 overexpression（n=20）. (B) Representative Western blots and analysis of Rasal3 (C), Cleaved caspase-3 (D) and BAX (E) in ALKBH5$^{flox/flox}$ and ALKBH5$^{flox/flox,\ \alpha\text{-}MyHC\text{-}Cre}$ after pAAV-OE-Rasal3 overexpression（n=3）. (F) Count of TUNEL in ALKBH5$^{flox/flox}$ and ALKBH5$^{flox/flox,\ \alpha\text{-}MyHC\text{-}Cre}$ mice after pAAV-OE-

Rasal3 overexpression (n=6）. (G) Apoptosis measured by TUNEL staining in $ALKBH5^{flox/flox}$ and $ALKBH5^{flox/flox, \alpha\text{-}MyHC\text{-}Cre}$ heart sections (Bar=80 μm). (H) Western blot analysis of Cleaved caspase-3 and BAX in ALKBH5-KO-CM-DOX and WT-CM-DOX after pAAV-shRNA-Rasal3 overexpression. Data are depicted as the mean ± SEM. Statistical significance was determined by student's t test or one-way ANOVA or two-way ANOVA with a post-hoc Holm-Sidak test, ns, not significant; *P<0.05; **P<0.05; ***P<0.001; ****P<0.0001.

Figure S17

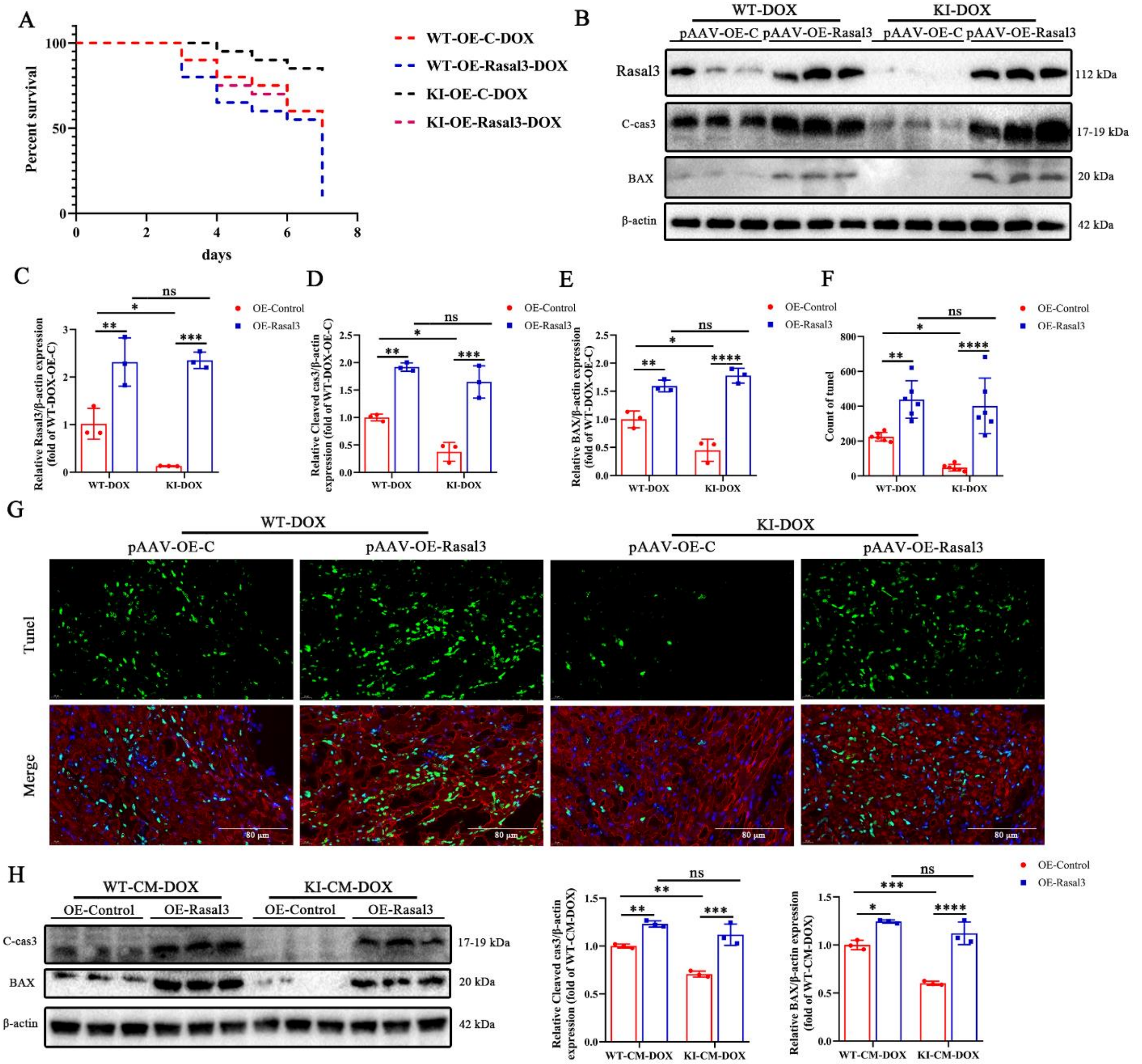

Figure S17. Rasal3 adenovirus overexpression aggravates DIC injury in ALKBH5-KI Mice. Related to the Figure 7.

(A) Kaplan Meier survival curves showing the survival of DOX-stressed mice in WT and KI mice after pAAV-OE-Rasal3 overexpression (n=20）. (B) Representative Western blots and analysis of Rasal3 (C), Cleaved caspase-3 (D) and BAX (E) in WT and KI mice after pAAV-OE-Rasal3 overexpression (n=3）. (F) Count of TUNEL in WT and KI mice after pAAV-OE-Rasal3 overexpression (n=6）. (G) Apoptosis measured by TUNEL staining in WT and KI mice heart

sections (Bar=80 μm). (H) Western blot analysis of Cleaved caspase-3 and BAX in ALKBH5-KI-CM-DOX and WT-CM-DOX after pAAV-shRNA-Rasal3 overexpression. Data are depicted as the mean ± SEM. Statistical significance was determined by student's t test or one-way ANOVA or two-way ANOVA with a post-hoc Holm-Sidak test, ns, not significant; *P<0.05; **P<0.05; ***P<0.001; ****P<0.0001.

Table S1. Mouse primers used in this study. Related to the Figure 1.

Gene	Forward primers (5'–3')	Reverse primers (5'–3')
METTL3	CTGGGCACTTGGATTTAAGGAA	TGAGAGGTGGTGTAGCAACTT
METTL14	CTGAGAGTGCGGATAGCATTG	GAGCAGATGTATCATAGGAAGCC
ALKBH5	CGCGGTCATCAACGACTACC	ATGGGCTTGAACTGGAACTTG
FTO	TTCATGCTGGATGACCTCAATG	GCCAACTGACAGCGTTCTAAG
β-actin	GGCTGTATTCCCCTCCATCG	CCAGTTGGTAACAATGCCATGT

Table S2. Mouse primers used in this study. Related to the Figure 5.

Gene	Forward primers (5'–3')	Reverse primers (5'–3')
Rasal3	TCCTGAGGCTTTTATGCCCTG	GGCGATGAGTGTAAGAGTTCTG
Mnda	GACAACCAAGAGCAATACACCA	ATCAGTTTGCCCAATCCAGAAT
F10	AGGACTCGGAGGGCAAACT	TCACGGACCTCTTCATAAGAACA
Phf11a	AGAGGGTCAAGAACCCACACA	TGACTCTGAGGCAGTCTCATT
Slfn8	TCCCCCGAGCATCCCTAAA	AGGGTGCCTGATATACTTTGGG
Crispld2	TGTGCAGTCATGGTACGACG	CAGCCGATCTTGTTGGTGGT
Slc40a1	GCGATCACAATCCAAAGGGAC	TTGGTTAGCTGGTCAATCCTTC
Cxcr2	ATGCCCTCTATTCTGCCAGAT	GTGCTCCGGTTGTATAAGATGAC
Uhmk1	CTCTCCCAATGTGCCATCAC	TTCGTGGTTTGAGGTCTGCAT
Itgal	CCAGACTTTTGCTACTGGGAC	GCTTGTTCGGCAGTGATAGAG
Megf6	CGTGCCAATGTCCTGGTGA	GACAGGCAAGGCTACAGTCG
Fv1	ACCCTGGAGTGGTATTCTATTGA	AGTCTCTACAGTTTCCCGGAC
Arhgap8	CTACGAGCAGATTCTCGGGAT	GCGGAGTACGGCATAGTTGT
β-actin	GGCTGTATTCCCCTCCATCG	CCAGTTGGTAACAATGCCATGT

Table S3. gRNA sequence. Related to the Figure 5.

gRNA	Guide RNA sequence (5’-3’)
gRNA-1	AATTAGGTATGAATTTGGGAGA
gRNA-2	TAATCATTTGTAGGGGCAGAGAA
gRNA-3	GTGACAACCTTAGATCACACCA

Expanded Materials 1: ALKBH5-KO mice design and identification results.

ALKBH5-KO

Aim	Purchase	Name	Technology	Target	Strain	Sample Source
ALKBH5-KO	GemPharmatech	B6/JGpt-Alkbh5em2Cd6815in34/Gpt	CRISPR/Cas9	ALKBH5-201	C57BL/6J Gpt	Tail

Technical routes: The ALKBH5 gene has 2 transcripts. According to the structure of ALKBH5 gene, exon1 of ALKBH5-201 (ENSMUST00000044250.3) transcript is recommended as the knockout region. The region contains start codon ATG. Knock out the region will result in disruption of protein function. In this project we use CRISPR/Cas9 technology to modify ALKBH5 gene. The brief process is as follows: sgRNA was transcribed in vitro. Cas9 and sgRNA were microinjected into the fertilized eggs of C57BL/6J mice. Fertilized eggs were transplanted to obtain positive F0 mice which were confirmed by PCR and sequencing. A stable F1 generation mouse model was obtained by mating positive F0 generation mice with C57BL/6J mice.

Alkbh5 alkB homolog 5, RNA demethylase [Mus musculus (house mouse)]

Gene ID: 268420, updated on 2-Mar-2019

Summary

Official Symbol Alkbh5 provided by MGI
Official Full Name alkB homolog 5, RNA demethylase provided by MGI
Primary source MGI:MGI:2144489
See related Ensembl:ENSMUSG00000042650
Gene type protein coding
RefSeq status VALIDATED
Organism Mus musculus
Lineage Eukaryota; Metazoa; Chordata; Craniata; Vertebrata; Euteleostomi; Mammalia; Eutheria; Euarchontoglires; Glires; Rodentia; Myomorpha; Muroidea; Muridae; Murinae; Mus; Mus
Also known as AW050020, AW215868, Abh5, E130207K11, Ofoxd
Expression Ubiquitous expression in adrenal adult (RPKM 20.2), ovary adult (RPKM 17.5) and 28 other tissues See more
Orthologs human all

The gene has 2 transcripts,all transcripts are shown below:

Name	Transcript ID	bp	Protein	Biotype	CCDS	UniProt	Flags
Alkbh5-201	ENSMUST00000044250.3	5730	395aa	Protein coding	CCDS24794	Q3TSG4	TSL:1 GENCODE basic APPRIS P
Alkbh5-202	ENSMUST00000134770.1	624	No protein	Processed transcript	-	-	TSL:3

The strategy is based on the design of Alkbh5-201 transcript,The transcription is shown below:

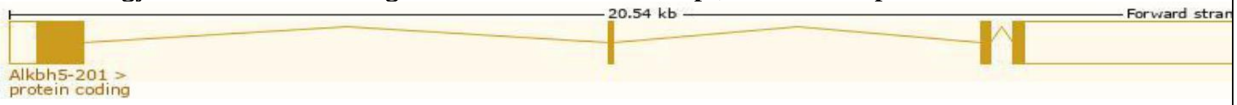

Prime

Primer Name	Sequence（5’-3’）	PCR size	Primer illustration
ALKBH5-WT-F	GACAGCAAGGATATGGGCCAAT	WT=252bp	Identify WT allele
ALKBH5-WT-R	CCCATATTAGGCTGGCACTTCT		
ALKBH5-KO-F	TGGATTACCACCAACACGAATGG	KO=683bp	Identify KO allele
ALKBH5-KO-R	GCTCCAGCTTCACGAGTTTGAG		

Gel electrophoresis

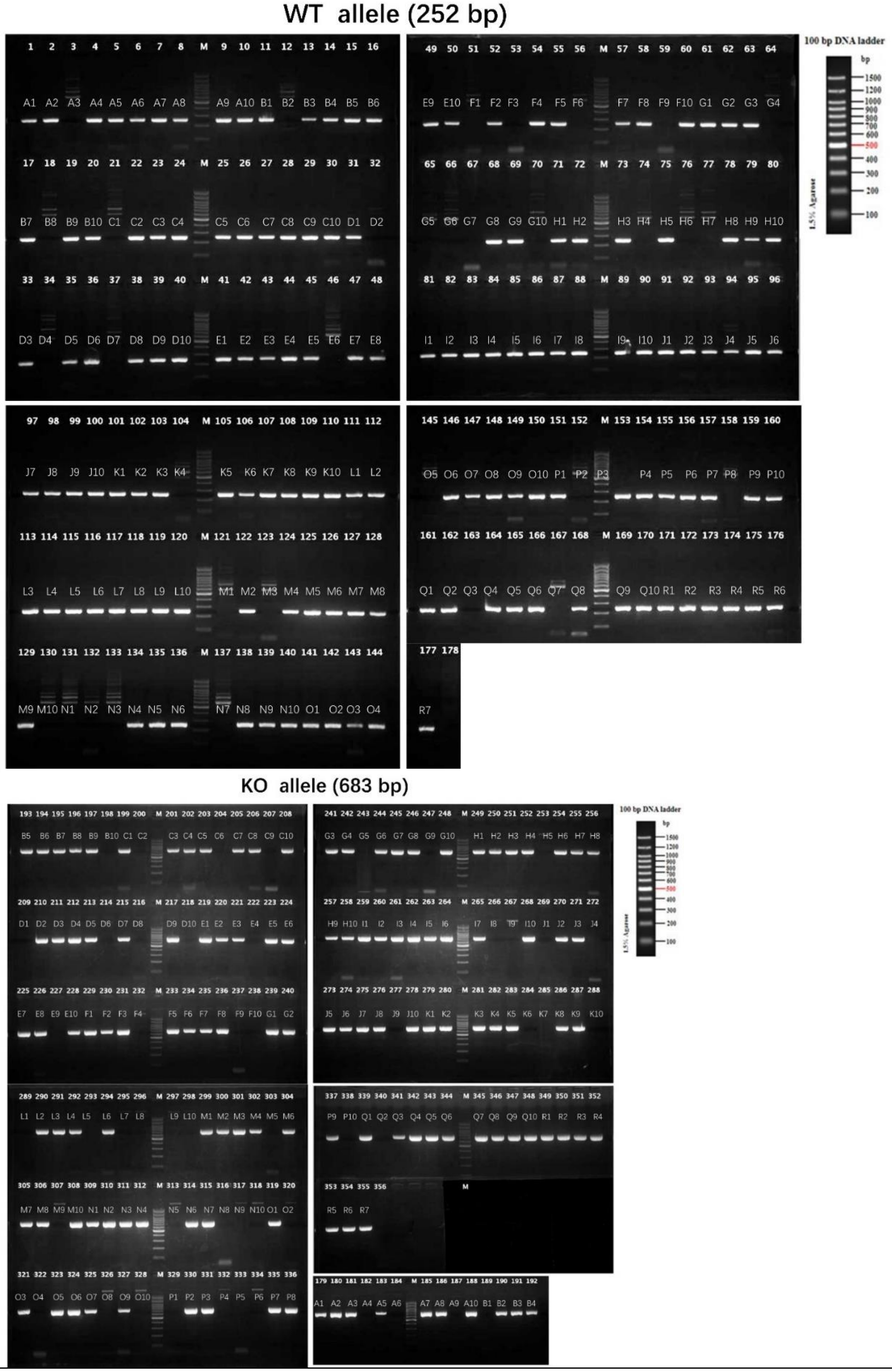

PCR Progress

**Reaction Components**	**Volume （μL）**
gDNA Template	2.0
10×*Taq* Buffer(mg^{2+} plus)	2.0
dNTP Mixture（10 mM）	0.5
Primer mix（10 μM）	0.5
Taq DNA polymerase(5 U/μL)	0.5
Milli-Q H_2O	To 20μL

**Seg.**	**Temp.**	**Time**	**Cycle**
1	95 ^oC	5min	
2	95 ^oC	30s	
3	58 ^oC	30s	
4	72 ^oC	30s	2-4,40
5	72 ^oC	3min	
6	25 ^oC	hold	

Expanded Materials 2: ALKBH5-KI mice design and identification results.

ALKBH5-KI

Aim	Purchase	Name	Technology	Target	Strain	Sample Source
ALKBH5-KI	GemPharmatech	B6/JGpt-H11em1Cin(CAG-ALKBH5-P2A-Luc-polyA)/Gpt	CRISPR/Cas9	ALKBH5-201	C57BL/6J Gpt	Tail

Technical routes:

3.1 Vector construction

The gRNA was designed, constructed and transcribed in vitro, and the homologous recombination vector (CAG-ALKBH5-P2A-Luc-polyA donor vector) to verify the correctness of the vector sequence by sequencing.

3.2 Microinjection

The Cas9, gRNA and Donor vector samples were microinjected into the fertilized eggs of mice with C57BL/6J background; The fertilized eggs that survived the injection were transplanted into pseudo-pregnant female mice, and they were pregnant and gave birth. 3.3 Identification of F0 generation mice The F0 generation pups born from the recipient mice have their tails and toes numbered in 5-7 days, and genomic DNA is extracted for PCR, Sequencing and southern blot identification to confirm the genotype.

3.4 Breeding of positive F0 generation mice

After the positive F0 generation mice become sexually mature, they will mate with wild-type background mice; The F1 generation mice born after 5-7 days of tail trimming and toe numbering, extraction of genomic DNA for PCR and sequencing Identification and confirmation of genotype.

Alkbh5 alkB homolog 5, RNA demethylase [Mus musculus (house mouse)]

Gene ID: 268420, updated on 2-Mar-2019

Summary

**Official Symbol**	Alkbh5 provided by MGI
**Official Full Name**	alkB homolog 5, RNA demethylase provided by MGI
**Primary source**	MGI:MGI:2144489
**See related**	Ensembl:ENSMUSG00000042650
**Gene type**	protein coding
**RefSeq status**	VALIDATED
**Organism**	Mus musculus
**Lineage**	Eukaryota; Metazoa; Chordata; Craniata; Vertebrata; Euteleostomi; Mammalia; Eutheria; Euarchontoglires; Glires; Rodentia; Myomorpha; Muroidea; Muridae; Murinae; Mus; Mus
**Also known as**	AW050020, AW215868, Abh5, E130207K11, Ofoxd
**Expression**	Ubiquitous expression in adrenal adult (RPKM 20.2), ovary adult (RPKM 17.5) and 28 other tissues See more
**Orthologs**	human all

The gene has 2 transcripts,all transcripts are shown below:

Name	Transcript ID	bp	Protein	Biotype	CCDS	UniProt	Flags
**Alkbh5-201**	ENSMUST00000044250.3	5730	395aa	Protein coding	CCDS24794	Q3TSG4	TSL:1 GENCODE basic APPRIS P1
**Alkbh5-202**	ENSMUST00000134770.1	624	No protein	Processed transcript	-	-	TSL:3

The strategy is based on the design of Alkbh5-201 transcript,The transcription is shown below:

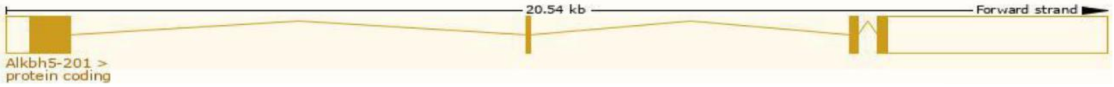

gRNA name	gRNA sequence（5’→3’）	PAM
gRNA1	CTGAGCCAACAGTGGTAGTA	AGG

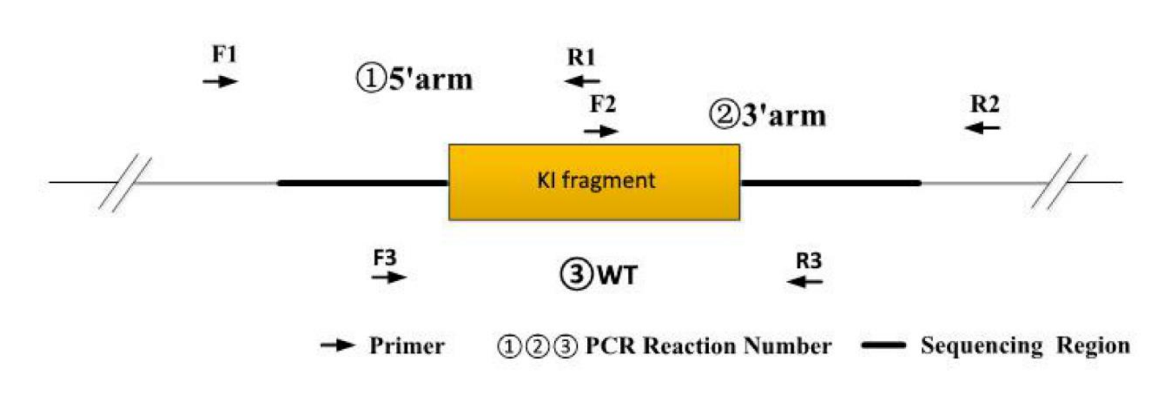

Prime	Primer Name	Sequence（5’-3’）	PCR size	Primer illustration
	H11-tF1	TCAGCGTTCAGACTCCTCAGAATGT	KI=1701bp<br>WT=none	Identify KI- 5’arm
	H11-CAG-5tR1	TCAATGGAAAGTCCCTATTGGCGT		
	H11-PolyA-3tF1	TGGCTGCCATGAACAAAGGTTG	KI=1491bp<br>WT=none	Identify KI- 3’arm
	H11-tR1	GACCCACTATTCTGCACCACTCATTAG		
	H11-WT-tF1	CAGCAAAACCTGGCTGTGGATC	WT=412bp	Identify WT allele
	H11-WT-tR1	ATGAGCCACCATGTGGGTGTC		

Gel electrophoresis

KI allele (1491 bp)

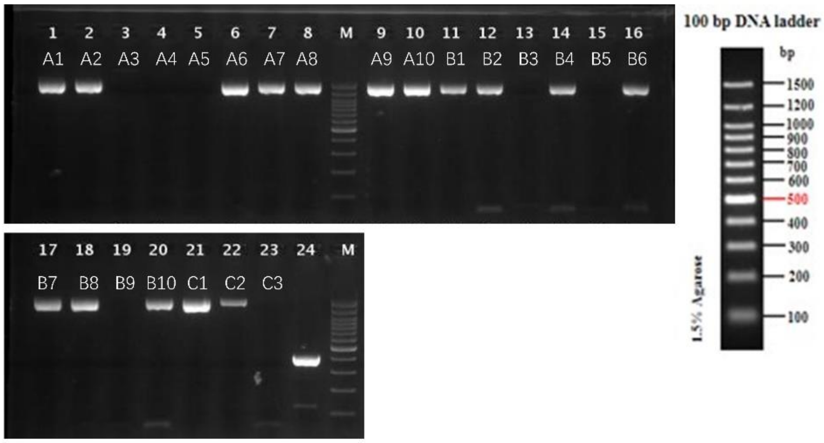

WT allele (412 bp)

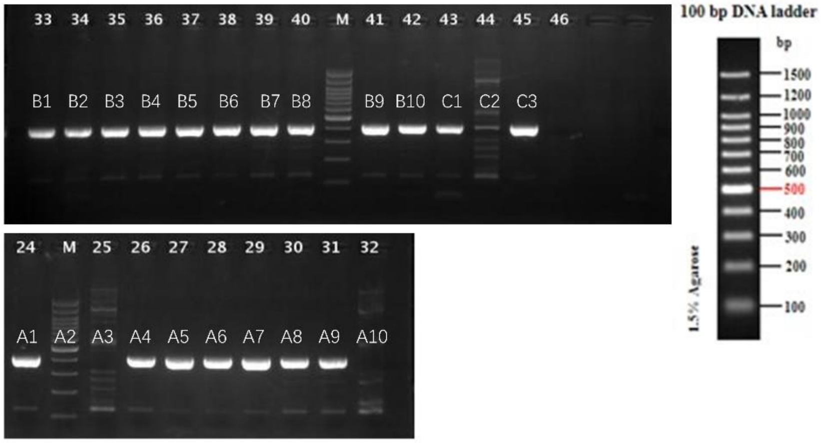

PCR Progress

**Reaction Components**	**Volume （μL）**
gDNA Template	2.0
10×*Taq* Buffer(mg^{2+} plus)	2.0
dNTP Mixture（10 mM）	0.5
Primer mix（10 μM）	0.5
Taq DNA polymerase(5 U/μL)	0.5
Milli-Q H_2O	To 20μL

**Seg.**	**Temp.**	**Time**	**Cycle**
1	95 °C	5min	
2	95 °C	30s	
3	58 °C	30s	
4	72 °C	30s	2-4,40
5	72 °C	3min	
6	25 °C	hold	

Expanded Materials 3: ALKBH5-myocardial specific knockout (ALKBH5$^{flox/flox,\ \alpha MyHC\text{-}Cre}$) mice design and identification results.

1. Product Information

Name	C57BL/6N-Alkbh5^{em1cyagen}
Serial Number	CKOCMP-268420-Alkbh5-B6N-VA
Gene	Alkbh5
NCBI ID	268420
Strain	C57BL/6N
Type	conditional knockout

2. Targeting Strategy

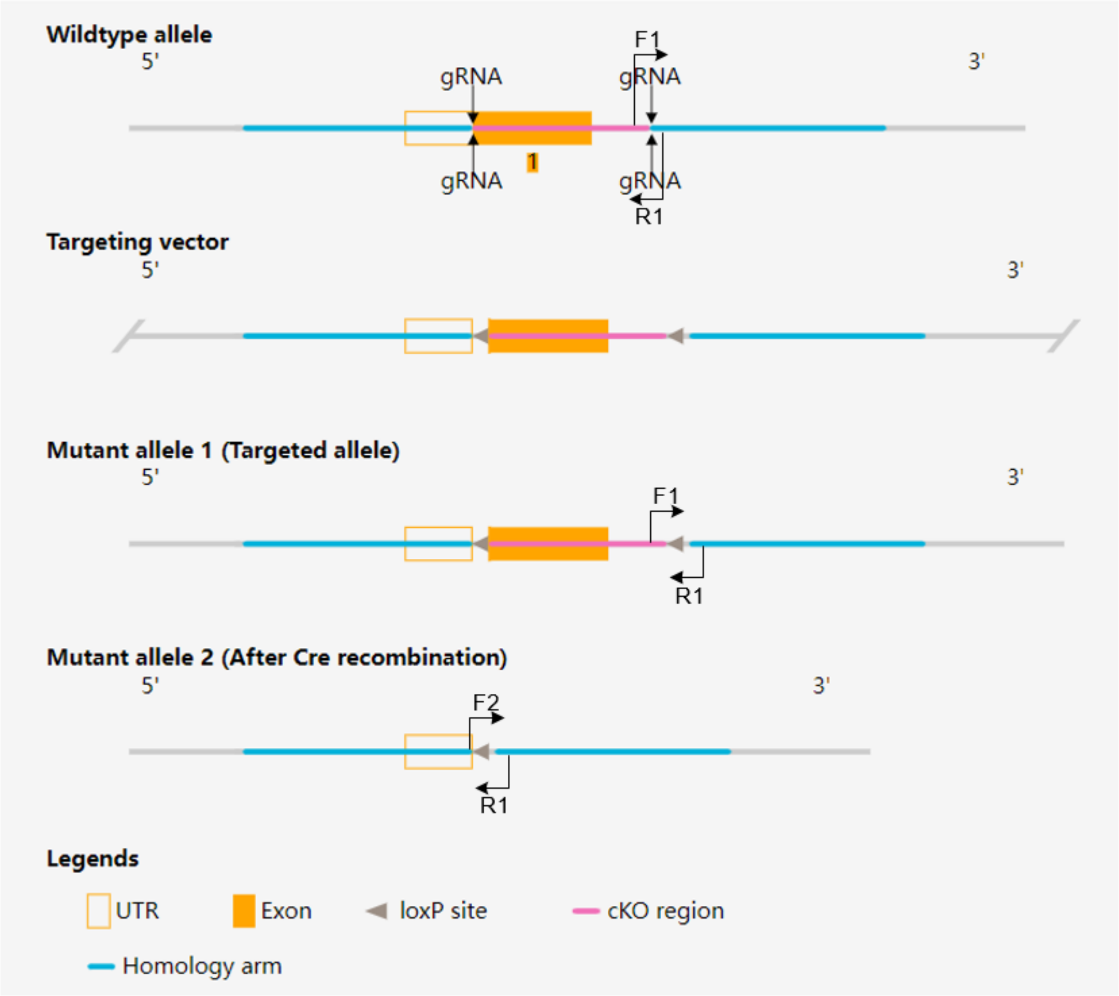

3. Genotyping strategy

Primers1: (Annealing Temperature 60.0 ºC)

F1: 5’-CTGAAGACTAGCATAGGAGTTCGAG-3’
R1: 5’-CCAAGACAGGAGAATCAGACATTCA-3’
Homozygotes: one band with 243 bp
Heterozygotes: two bands with 243 bp and 170 bp
Wildtype allele: one band with 170 bp

Primers2: (Annealing Temperature 60.0 ºC)

αMyHC-M-F : 5’-ATGACAGACAGATCCCTCCTATCTCC-3’
αMyHC-M-R : 5’-CTCATCACTCGTTGCATCATCGAC-3’
Creamplicon:~300 bp

The tissue-specific gene deletion can be confirmed by adding one additional primer to the PCR assay:
Primers3: (Annealing Temperature 60.0 ºC)

F2: 5’-GGAGGACCCTAGAGTTGAGTTG-3’
R1: 5’-CCAAGACAGGAGAATCAGACATTCA-3’
With Cre activity: one band with 142 bp

4. Expected Results

Genotyping	Primers1	Primers2
Flox/flox:<br>Homozygotes	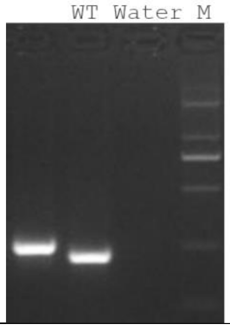	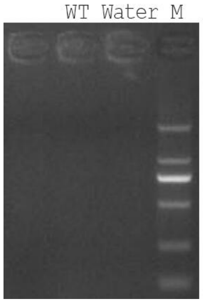
Flox/flox, αMyHC-Cre:<br>Homozygotes, Cre	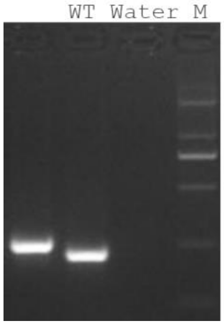	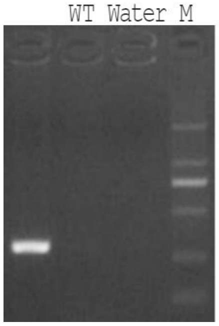

Note:

1) PCR was carried out in 25 μL volume for 35 cycles under standard conditions, with all two primers listed above added to each reaction.

2) DNAmarker:Thermo Scientific GeneRuler 2000 bp DNA Ladder #MK001

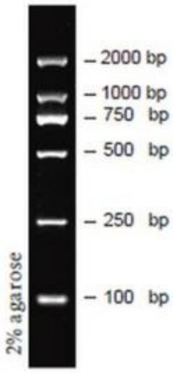

3) Controls used in PCR genotyping are:

- Water control: No DNA template added.
- Wildtype control: Mouse genomic DNA.

5. PCR reaction

5.1 DNA Extraction

- Method One:

We recommend that using TaKaRaMiniBEST Universal Genomic DNA Extraction kit (Ver.5.0_Code No. 9765) to gain high purity of genomic DNA.

a. Add 180 μL of Buffer GL, 20 μL of Proteinase K and 10 μL of RNase A per tail piece (2-5 mm) in a microcentrifuge tube. Be careful not to cut too much tail.

b. Incubate the tube at 56℃ overnight.

c. Spin in microcentrifuge at 12,000 rpm for 2 minutes to remove impurities.

d. Add 200 μL Buffer GB and 200 μL absolute ethyl alcohol with sufficient mixing.

e. Place the spin Column in a collection tube. Apply the sample to the spin and centrifuge at 12,000 rpm for 2 min. Discard flow-through.

f. Add 500 μL Buffer WA to the spin column and centrifuge at 12,000 rpm for 1 min. Discard flow-through.

g. Add 700 μL Buffer WB to the spin column and centrifuge at 12,000 rpm for 1 min. Discard flow-through. (Note: Make sure the Buffer WB has been premixed with 100% ethanol. When adding Buffer WB, add to the tube wall to wash off the residual salt.)

h. Repeat step g.

i. Place the spin Column in a collection tube and centrifuge at 12,000 rpm for 2 min.

j. Place the spin Column in a new 1.5ml tube. Add 50~200 μL sterilized water or elution buffer to the center of the column membrane and let the column stand 5min. (Note: Heating sterilized water or elution buffer up to 65℃ can increase the yield of elution.)

k. To elute DNA, centrifuge the column at 12,000 rpm for 2 min. To increase the yield of DNA, add the flow-through and/or 50~200 μL sterilized water or elution buffer to the center of the spin column membrane and let the column stand 5 min. Centrifuge at 12,000 rpm for 2 min.

l. Quantify to genomic DNA. Eluted genomic DNA can be quantified by electrophoresis or electrophoresis.

- Method Two:

A low-cost and sample method to gain rough genomic DNA.

a. Add 100 μL of tail digestion buffer per tail piece (2-5 mm) in a microcentrifuge tube. Be careful not to cut too much tail.

b. Incubate the tube at 56℃ overnight.

c. Incubate the tube at 98℃ for 13 minutes to denature the Proteinase K.

d. Spin in microcentrifuge at top speed for 15 minutes. Use an aliquot of supernatant straight from the tube (1 μL in a 12.5 μL reaction) for PCR.

Final concentration of tail digestion buffer:

- 50 mMKCl
- 10 mM Tris-HCl (pH 9.0)
- 0.1 % Triton X-100
- 0.4 mg/mL Proteinase K

5.2 PCR Mixture (primer concentration: 10µM):

Component	x1	
ddH_2O	9.0	µl
Product primer F	1.0	µl
Product primer R	1.0	µl
Premix Taq	12.5	µl
DNA	1.5	µl
Total	25	µl

5.3 PCR Reaction Conditions:

Step	Temp.	Time	Cycles
Initial denaturation	94 °C	3 min	
Denaturation	94 °C	30 s	35 x
Annealing	60 °C	35 s	
Extension	72 °C	35 s	
Additional extension	72 °C	5 min	

5.4 Relevant Reagents:

**TrizmaHydrochloride Solution**	Sigma, Cat. No. T2663
**Proteinase K**	Merck, Cat. No. MK539480
**Triton X-100**	Sigma, T8787-50 mL
**2 × Taq Master Mix (Dye Plus)**	Vazyme, P222
**Agarose**	BIOWEST AGAROSE, REGULAR
**DNA Marker**	Thermo Scientific GeneRuler 2000 bp DNA Ladder #MK001
**0.5×TBE**	Tris Bio Basic Inc, TBO194-500g
	EDTA Shanghai Sangon, 0105-500g
	Boric Acid, Shanghai Sangon, 0588-500g
